# Exploring fecal microbiota signatures associated with immune response and antibiotic impact in NSCLC: insights from metagenomic and machine learning approaches

**DOI:** 10.3389/fcimb.2025.1591076

**Published:** 2025-07-28

**Authors:** Wenjie Han, Yuhang Zhou, Yiwen Wang, Xiaolin Liu, Tao Sun, Junnan Xu

**Affiliations:** ^1^ Department of Breast Medicine 1, Cancer Hospital of China Medical University, Liaoning Cancer Hospital, Shenyang, China; ^2^ Department of Pharmacology, Cancer Hospital of China Medical University, Liaoning Cancer Hospital, Shenyang, China; ^3^ Department of Bioinformatics, Kanghui Biotechnology Co., Ltd., Shenyang, China; ^4^ Department of Breast Medicine, Cancer Hospital of Dalian University of Technology, Liaoning Cancer Hospital, Shenyang, China

**Keywords:** metagenome, machine learning, immunotherapy, lung cancer, gut microbiota, antibiotics

## Abstract

**Background:**

Substantial interstudy heterogeneity in cancer immunotherapy-associated biomarkers has hindered their clinical applicability. To address this challenge, we performed a comprehensive integration of publicly available global metagenomic datasets. By leveraging metagenomic profiling and machine learning approaches, this study aimed to elucidate gut microbial signatures associated with immune response in lung cancer (LC) and to evaluate the modulatory effects of antibiotic exposure.

**Methods:**

A systematic literature search was conducted to identify relevant datasets, resulting in the inclusion of 209 fecal metagenomic samples: 154 baseline samples (45 responders, 37 non-responders, and 72 healthy controls) and 55 longitudinal samples collected during immunotherapy. We performed taxonomic and functional characterization of gut microbiota (GM) differentiating responders from non-responders, delineated microbiome dynamics during treatment, and assessed the impact of antibiotics on key microbial taxa. Among eight machine learning algorithms evaluated, the optimal model was selected to construct a predictive framework for immunotherapy response.

**Results:**

Microbial α-diversity was significantly elevated in responders compared to non-responders, with antibiotic administration further amplifying this difference—most notably at the species level. Integrative multi-omics analysis identified two pivotal microbial biomarkers, *s_Bacteroides caccae* and *s_Prevotella copri*, which were strongly associated with immunotherapy efficacy. A random forest-based classifier achieved robust predictive performance, with area under the curve (AUC) values of 0.82 and 0.79 at the species and genus levels, respectively. Notably, *P. copri* was further enriched in responders with poor progression-free survival (PFS <3 months), indicating a potential deleterious role. Antibiotic exposure significantly influenced the abundance and functional potential of these key taxa. KEGG-based functional analysis revealed the enrichment of amino acid metabolism pathways in responders. Additionally, CARD database annotation demonstrated that the majority of antibiotic resistance genes were associated with *Bacteroidetes* and *Proteobacteria*, implicating these taxa in shaping microbial-mediated therapeutic responses.

**Conclusions:**

This study represents the first large-scale, cross-cohort integration of metagenomic data to identify reproducible GM signatures predictive of immune checkpoint inhibitor efficacy in LC. The findings not only underscore the prognostic relevance of specific taxa but also establish a foundation for developing microbiome-informed, personalized immunotherapeutic strategies.

## 1 Introduction

Over the past decade, immune checkpoint inhibitors (ICIs) targeting PD-1/PD-L1 and CTLA-4 have revolutionized the therapeutic landscape of lung cancer (LC). However, only approximately 20% of patients with locally advanced or metastatic disease derive sustained clinical benefit from immunotherapy (Cascone et al., 2022; Diao et al., 2025; Huang et al., 2022). Therefore, identifying reliable predictive biomarkers and elucidating the mechanisms underlying responses to ICIs remain key scientific challenges in the field of tumor immunotherapy.

Although PD-L1 expression is commonly employed as a predictive biomarker in clinical practice, it has limited prognostic value in squamous cell carcinoma and exhibits significant spatial and temporal heterogeneity between primary and metastatic lesions (Doroshow et al., 2021; Hirsch et al., 2022). Similarly, tumor mutational burden (TMB) has been proposed as an alternative marker; however, the lack of consensus on threshold definitions and technical inconsistencies across sequencing platforms hinder its routine clinical application (Sholl et al., ). Emerging strategies, such as profiling the tumor immune microenvironment, analyzing circulating tumor DNA (ctDNA), and applying high-dimensional single-cell omics, hold promise, but their clinical utility is constrained by high cost, insufficient standardization, and technical complexity (Datar et al., 2019; Leng et al., 2021; Zhang et al., 2020). Therefore, there remains a pressing need to develop low-cost, non-invasive, and biologically stable biomarkers with a robust predictive value for immunotherapy response.

Our previous work demonstrated that LC progression is associated with significant alterations in the composition of the host-associated microbiota, suggesting a potential role of the gut microbiome in LC development and immune regulation (Han et al., 2023). The influence of the GM on cancer immunotherapy has garnered increasing attention in recent years. As early as 2007, murine models first demonstrated that gut microbes can activate antitumor immune responses, marking the inception of microbiota–immunity research (Paulos et al., 2007). In 2015, prospective studies further confirmed a direct association between gut microbial composition and ICI efficacy (Vétizou et al., 2015). In 2018, fecal microbiota transplantation (FMT) from responders was shown to enhance ICI sensitivity in recipient mice (Routy et al., 2018). By 2021, human-to-human FMT was successfully translated into clinical settings, offering a novel approach to overcome resistance to PD-1 blockade (Davar et al., 2021).

Several single-center studies conducted in South Korea, China, the United States, and France have investigated gut microbiome alterations associated with ICI response (Fang et al., 2022; Routy et al., 2018; Lee et al., 2021; Liu et al., 2022). However, the heterogeneity of findings across cohorts raises concerns regarding the reproducibility and translational applicability of candidate microbial biomarkers. Among them, *Akkermansia muciniphila* (AKK) has been repeatedly reported as a favorable taxon in responders and was highlighted as a top-ranked biomarker in a landmark study published in *Science* (Routy et al., 2018). Using spatial metabolomics, Zhu et al. demonstrated that *Akk* can translocate from the gut to the bloodstream, colonize lung tumor tissue, and exert antitumor effects by modulating tumor-associated microbiota and reprogramming tumor metabolism (Zhu et al., 2023). However, contrasting findings by Lee et al. reported the enrichment of *Akk* in non-responders, further underscoring inconsistencies in the current literature (Lee et al., 2021).

To date, no large-scale, multi-cohort meta-analysis has systematically evaluated GM signatures associated with ICI response in LC. To address this gap, we conducted an integrative analysis of publicly available metagenomic datasets from multiple countries and regions. Using a standardized metagenomic pipeline and machine learning-based modeling framework, we aimed to identify reproducible microbial features strongly associated with immunotherapy response and to discover stable and clinically translatable microbial biomarkers. Our findings provide critical insights into the microbiota–immune axis and establish a foundation for microbiome-informed personalized immunotherapy strategies in LC.

## 2 Methods

### 2.1 Data acquisition and mining

The literature search strategy and the PRISMA flow diagram are detailed in Section 1 of the **Supplementary Materials**. Five relevant studies were identified, corresponding to the following NGS project accession numbers: PRJNA866654, PRJEB26531, PRJNA494824, PRJEB22863, and PRJNA48479 (Human Microbiome Project). The inclusion and exclusion criteria for cohort selection are provided in Section 1.3 of the **Supplementary Materials**. The quality of this meta-analysis was evaluated using the ROBIS tool and the Joanna Briggs Institute Critical Appraisal Checklist for Case–Control Studies. The detailed assessment results are provided in Section 2 of the **Supplementary Materials**.

### 2.2 Shotgun metagenomics sequencing and bioinformatics analysis

#### 2.2.1 Metagenomic data processing and gene catalog construction

Raw sequencing data were subjected to quality control using Fastp (v0.21.0) to remove low-quality bases, low-quality reads, and residual sequencing adapters (Chen et al., 2018). Clumpify (v38.90) was then applied to eliminate duplicate reads. To filter out host-derived sequences, Bowtie2 (v2.4.2) was used with default parameters to align reads against the human reference genome (GRCh38.p13), thereby generating high-quality, host-depleted clean data (Li et al., 2019). Subsequently, MEGAHIT was employed for the metagenomic assembly of the clean reads, and contigs shorter than 500 bp were removed using QUAST for quality assessment and filtration. Gene prediction was performed on the assembled contigs using MetaGeneMark. The resulting gene set was clustered and de-redundified using CD-HIT with a sequence identity threshold of 95% and a coverage cutoff of 90%. The longest sequence from each cluster was retained as a representative unigene. Gene abundance for each sample was quantified using Salmon based on the alignment of clean reads to the non-redundant unigene catalogue.

#### 2.2.2 Taxonomic profiling and microbial community analysis

Taxonomic annotation was performed using MetaPhlAn4 with default parameters to generate microbial profiles at multiple taxonomic levels. Alpha diversity metrics, including species richness and Shannon index, were calculated using the VEGAN R package (v2.5.3). Beta-diversity was assessed based on Bray–Curtis dissimilarity and visualized via principal coordinate analysis (PCoA). Differences in microbial composition between groups were evaluated using the analysis of similarities (ANOSIM). To identify differentially abundant taxa, linear discriminant analysis effect size (LEfSe) was conducted with an LDA score threshold of >4.0. Co-occurrence and clustering analyses were performed using the R packages COOCCUR and psych, applying a significance threshold of *p* < 0.05 and a Pearson correlation coefficient ≥0.3. The resulting networks were visualized in Cytoscape (v3.10.1). Due to the high resolution of mNGS-based taxonomic profiling, which yielded annotations for over 180,000 species and 3,000 genera, only the top 50 most abundant species and genera were retained for co-occurrence network construction to ensure interpretability and visualization clarity.

#### 2.2.3 Confounder analysis

To assess the influence of potential confounding variables on individual microbial taxa within LC microbiome studies related to immune response, we adopted a variance decomposition strategy inspired by the approach of Jakob Wirbel et al. (Wirbel et al., 2019). Specifically, we partitioned the total variance in microbial abundance into components attributable to immune response status and to individual confounding factors. This framework parallels a linear modeling approach in which microbial abundance is modeled as a function of both immune response classification and the confounding variable. Given the non-normal distribution commonly observed in microbiome data, variance calculations were conducted using rank-transformed abundance values.

#### 2.2.4 Functional and antibiotic resistance profiling of unigenes

To functionally characterize unigenes, DIAMOND and HUMAnN3 were employed to align protein sequences against the Kyoto Encyclopedia of Genes and Genomes (KEGG) database using *blastp* with an E-value threshold of ≤1e-5. The alignment results were filtered to retain only the top-scoring hit per query, defined as a high-scoring pair (HSP) with a bit score >60. For HUMAnN3 outputs, the best-scoring HSP (>60 bits) was similarly selected for downstream analysis. Functional pathway enrichment analysis was performed using the Reporter Score algorithm, with enrichment deemed statistically significant when the absolute score exceeded the 95% confidence interval (i.e., |score| > 1.96). Additionally, unigenes were annotated against the Comprehensive Antibiotic Resistance Database (CARD) using the Resistance Gene Identifier (RGI) tool (*blastp*, E-value ≤1e-30). The resulting Antibiotic Resistance Ontology (ARO) abundance profiles were used to assess differential abundance between experimental groups and to infer the taxonomic origin of resistance genes.

#### 2.2.5 Development and validation of a machine learning-based predictive model

A total of eight machine learning algorithms, including random forest (RF), neural network (NNet), and Treebag, were systematically evaluated for model development. Based on the AUC and overall predictive performance, the RF algorithm was selected as the primary classifier in this study. Feature importance was quantified using the Gini index, and features were ranked accordingly to identify key predictive variables. Tenfold cross-validation was subsequently applied to the trained decision tree model to optimize feature selection and determine the optimal number of predictors.

## 3 Results

### 3.1 Confounding factors and batch effect control

A total of five published studies related to LC immunotherapy were selected from 645 articles, with the detailed characteristics of the meta-cohort summarized in **Table 1**. A total of 209 fecal samples were collected, including 154 baseline samples (responder = 45, non-responder = 37, and healthy = 72) and 55 samples during treatment. **Supplementary Tables S1**, **S2** respectively provide an overview of sample processing methods and diversity analysis results for each study. Additionally, a risk of bias assessment was conducted for the included studies, with findings presented in Section 2 of the **Supplementary Material**. The principal risk identified was insufficient identification and control of potential confounding factors in the original studies.

**Table 1:** Clinical characteristics of the cohorts included in the meta-analysis.

Study	First author	Year	Data type	Sample numbers	Age (average ± s.d.^a^)	Type of immunotherapy given	Accession number	Country
Study 1	Ben Liu (Liu et al., 2022)	2022	WGS	14	66.57 ± 12.95	Anti-PD-1	PRJNA866654	Iowa, USA
Study 2	Se-Hoon Lee (Lee et al., 2021)	2021	WGS	2	61.00 ± 7.07	Anti-PD-1	PRJEB26531	Seoul, Korea
Study 3	Yoshitaro Heshiki (Heshiki et al., 2020)	2020	WGS	3	53.63 ± 6.72	Anti-PD-1	PRJNA494824	Arizona, USA
Study 4	Bertrand Routy (Routy et al., 2018)	2018	WGS	118	62.81 ± 9.61	Anti-PD-1	PRJEB22863	Paris, France
Study 5	HMP^a^	2015	WGS	72	_	_	PRJNA48479	USA
Validation study 1	Rachel C. Newsome (Newsome et al., 2022)	2022	16s rRNA	65	51 ± 49	Anti-PD-1/CTLA-4, anti-PD-L1	PRJNA768678	Tampa, FL, USA

a The Human Microbiome Project (HMP) is a large-scale DNA sequencing program led by the National Institutes of Health. From it, we extracted metagenomic shotgun sequencing data from healthy subjects for downstream analysis (http://segatalab.cibio.unitn.it/tools/metaphlan2/).

**Figure 1:**
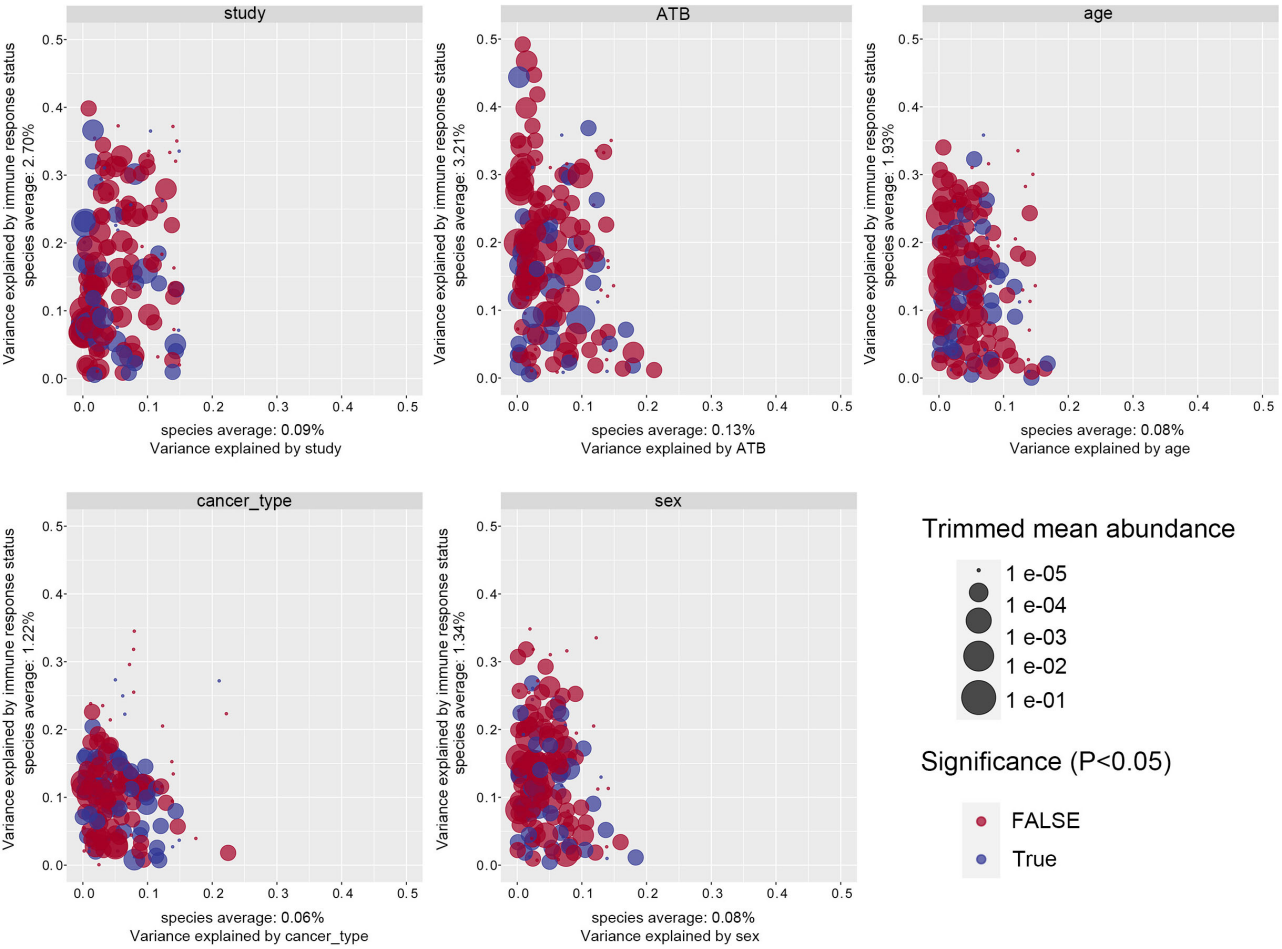
Variance explained by response status (response vs. non-response) is plotted against variance explained by the confounding factor effects for individual microbial species. The significantly differential microbiota are colored in blue, and *P*-values were from the two-way ANOVA test. The abundance of each taxa is plotted proportionally to the dot size. ANOVA, analysis of variance.

Considering the technical and biological heterogeneity inherent across studies, we evaluated the impact of confounding variables including study origin, antibiotic exposure, age, sex, and cancer subtype on gut microbiome composition. Immune response status consistently explained the largest proportion of microbial community variance, surpassing all other covariates, indicating that confounding effects were relatively minor compared to the biological signal of interest. Among clinical variables, antibiotic exposure accounted for the second largest variance component after immune status, exceeding the contributions of study origin, age, and other factors (**Figure 1**). To mitigate the influence of antibiotic exposure, the samples were stratified accordingly for downstream analyses. Study origin was identified as the second most significant confounder. To control for batch effects, study origin was incorporated as a blocking factor, and a two-sided blocked Wilcoxon rank-sum test was employed to identify differentially abundant taxa. Only taxa exhibiting minimal variance attributable to study origin were retained for further analysis.

### 3.2 Baseline GM taxonomic differences between responder and non-responder

In the following analyses, the study population was stratified into the total cohort, an antibiotic-exposed group (ATB), and a non-antibiotic-exposed group (non-ATB) to evaluate differences in gut microbiota between responders (R) and non-responders (NR) across subgroups and to assess the impact of antibiotic exposure on these differences.

**Figure 2:**
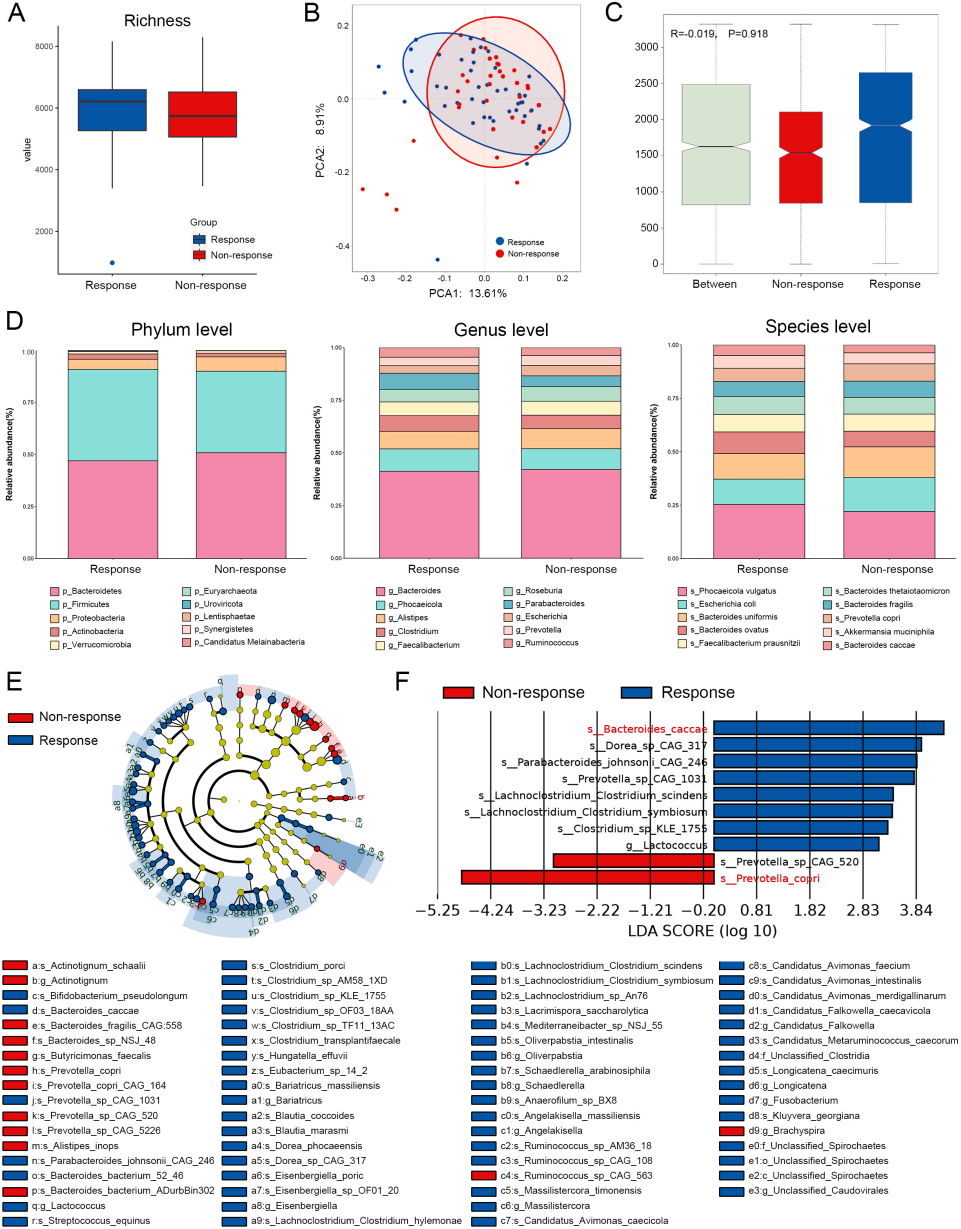
Species composition and difference analysis of response group and non-response group based on total population. **(A)** α-diversity analysis with richness as the index. **(B)** Baseline gut microbiota PCOA analysis results of the two groups. **(C)** Similarity analysis of baseline microbiota between the two groups. **(D)** Stacking maps of top 10 abundance at the phylum, genus, and species levels between responders and non-responders. **(E)** Phylogenetic branching map between the two groups based on the results of the difference analysis. **(F)** Results of LEfSe difference analysis.

In terms of species richness, the R group consistently exhibited a higher α-diversity compared to the NR group, and this disparity was further amplified in the presence of antibiotic exposure (**Figure 2A**, **Supplementary Figure S1**). Principal coordinates analysis (PCoA) and analysis of similarities (ANOSIM) indicated no statistically significant differences in β-diversity between the R and NR groups, although antibiotic exposure appeared to increase the compositional divergence. Notably, inconsistent findings on β-diversity reported across similar studies may stem from population heterogeneity, sample processing variability, and differences in sequencing platforms.

The results of the analysis of the top 10 taxa at different taxonomic levels (**Figure 2D**, **Supplementary Figures S2D**, **S3D**) revealed that *Firmicutes* and *Bacteroidetes* were the dominant phyla in both groups. At the genus level, *Bacteroides* consistently ranked as the most abundant genus across all subgroups, while the remaining top genera showed minimal abundance differences between the R and NR groups. However, antibiotic exposure substantially influenced the enrichment patterns of several dominant genera—for instance, *Phocaeicola*, *Faecalibacterium*, and *Roseburia* were relatively enriched in the R group within the non-ATB cohort but showed decreased abundance in the R group under antibiotic exposure, suggesting that antibiotics may disrupt the natural enrichment of beneficial bacteria. A similar trend was observed at the species level; however, certain taxa exhibited enrichment patterns that were not affected by antibiotic exposure, such as *Bacteroides uniformis* and *B. caccae*.

To further identify differential biomarkers between groups, we employed LEfSe. The cladogram-based visualization showed that most differences between the R and NR groups were concentrated at the species level (**Figure 2E**, **Supplementary Figures S2E**, **S3E**). The LEfSe analysis of the total cohort identified *B. caccae* and *P. copri* as the top-ranked biomarkers for the R and NR groups, respectively (**Figure 2F**). *B. caccae* was also selected as a key differential species in the non-ATB population (**Supplementary Figure S2F**).

### 3.3 Intra-community bacterial interactions in network relationships

**Figure 3:**
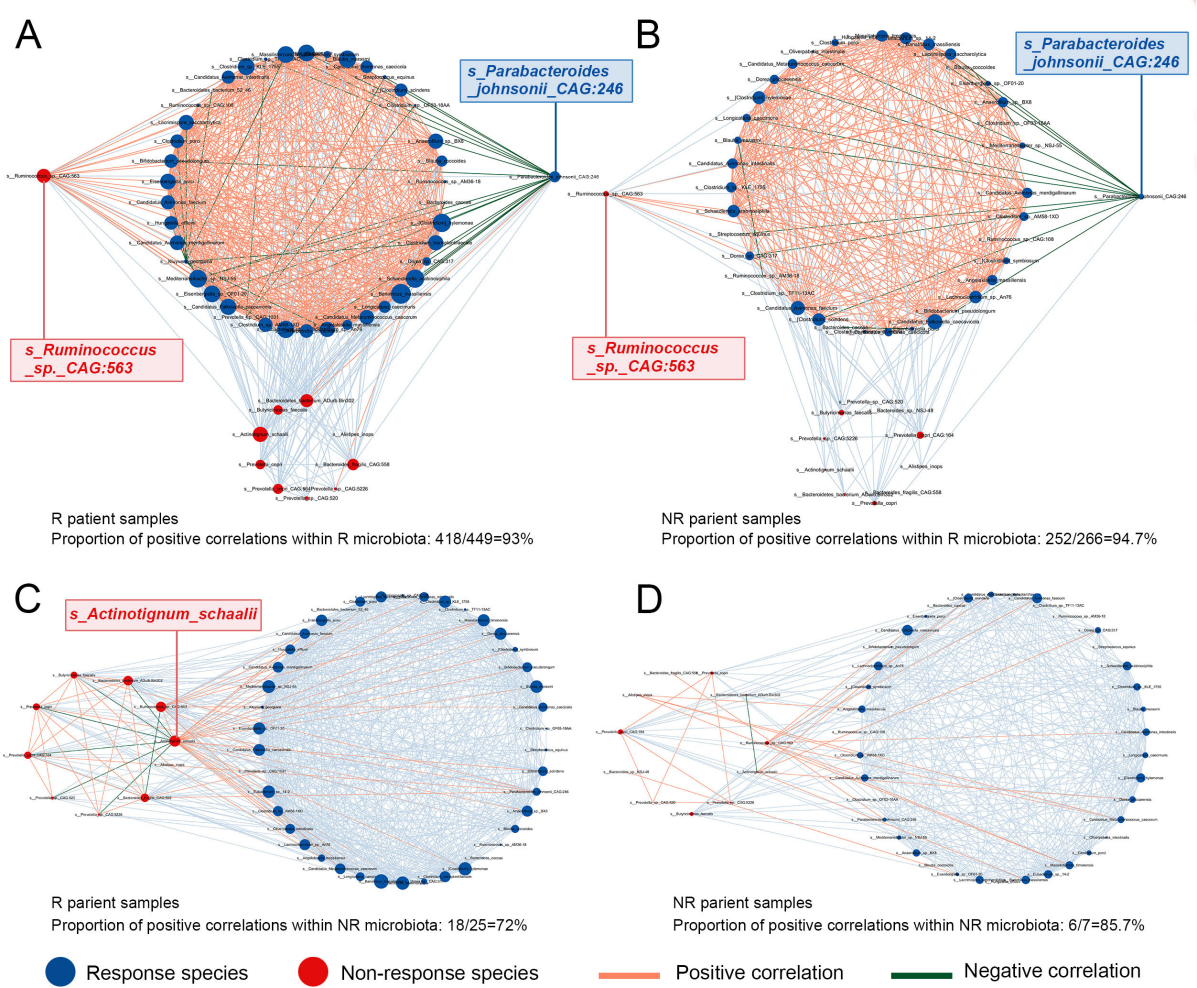
Interaction of differential markers between responder and non-responder groups within different populations. **(A)** Intra-community and inter-community interactions of the responder microbiota in the responder population. **(B)** Intra-community and inter-community interactions of responders in non-responders. **(C)** Intra-community and inter-community interactions of non-responsive bacteria in responders. **(D)** Intra-community and inter-community interactions of non-responsive bacteria in non-responsive population.

In both the responder (R) and non-responder (NR) populations, the intra-community positive correlations among species were consistently stronger within the R microbiota compared to the NR microbiota. However, the overall interaction strength within the R microbiota did not differ significantly between the R and NR populations (**Figures 3A, B**), whereas the NR microbiota displayed significantly stronger intra-community associations in the NR population than in the R population (**Figures 3C, D**).

Notably, in the R population, *s_Parabacteroides johnsonii_CAG:246* exhibited strong negative correlations with other R-associated taxa (**Figure 3A**), a pattern that was similarly observed in the NR population (**Figure 3B**). As a species enriched in responders, this bacterium appears to exert antagonistic effects on other beneficial taxa, suggesting a potentially suppressive role in immune modulation and highlighting its relevance as a candidate negative regulator of therapeutic response.

Conversely, *s_Ruminococcus_sp._CAG:563*, although enriched in the NR microbiota, showed strong positive associations with multiple R-associated taxa (**Figures 3A, B**). This suggests a possible synergistic role in enhancing the abundance or activity of beneficial microbes associated with immune response and raises the hypothesis that supplementation with this species might augment treatment efficacy.

Within the NR microbiota, an intriguing pattern emerged: *s_Actinotignum_schaalii* was strongly negatively correlated with other NR-associated species while demonstrating robust positive correlations with R-associated taxa (**Figure 3C**). Although not enriched in the R population, this species may simultaneously suppress non-beneficial taxa and promote beneficial ones, potentially contributing to improved immune responsiveness via a dual mechanism. The immunomodulatory roles—either tumor-promoting or tumor-suppressive—of these taxa warrant further investigation through functional studies.

### 3.4 Construction and validation of machine learning model for immunotherapy effect

**Figure 4:**
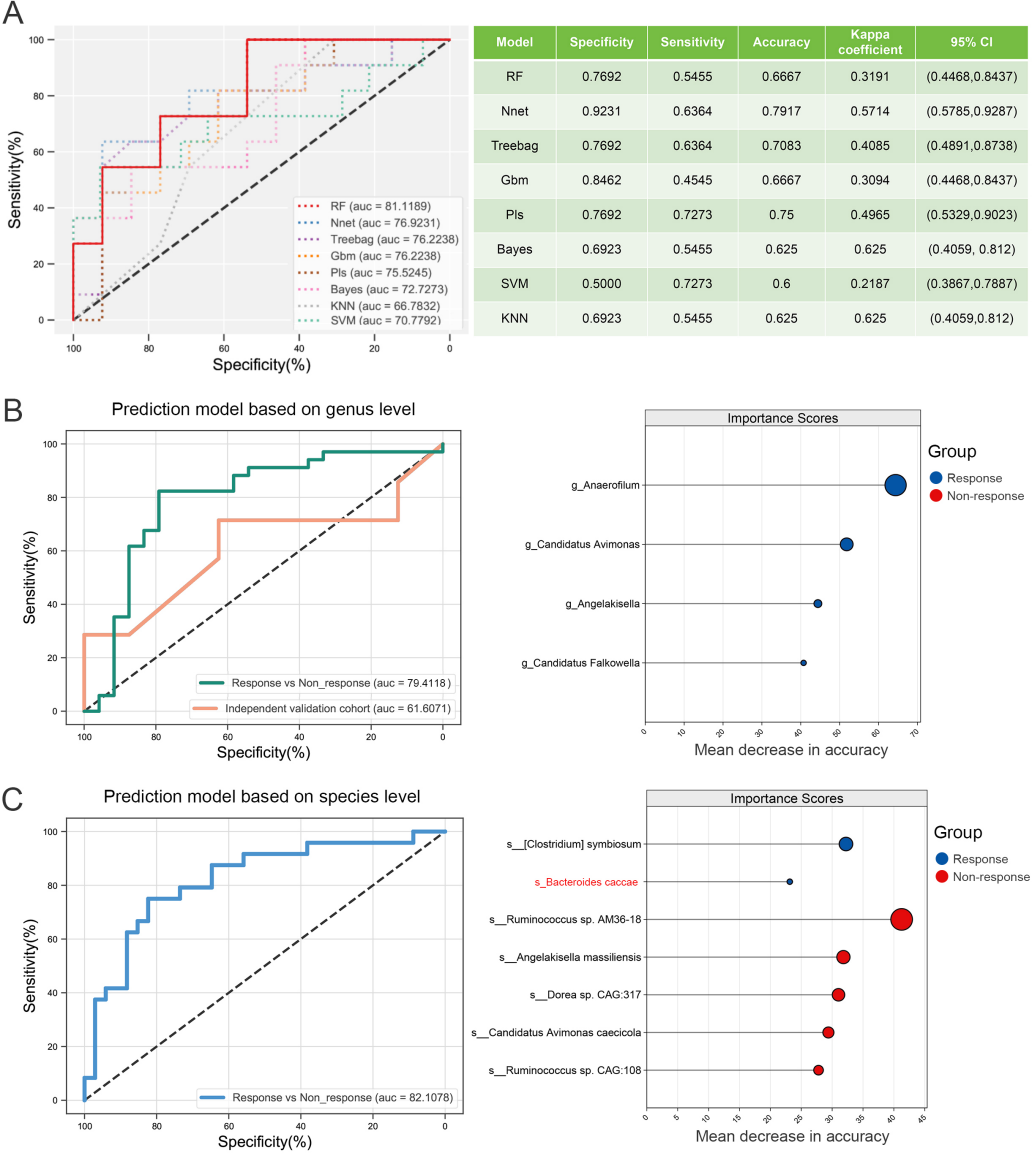
Screening and construction of immune efficacy prediction models. **(A)** Comparison of prediction efficiency and performance of different machine learning methods. **(B)** The RF model was established and validated based on the genus-level differential markers obtained by LEfSe analysis. **(C)** RF modeling was performed at the species level based on the differential species obtained by LEfSe analysis.

To optimize predictive performance, we evaluated eight widely used machine learning algorithms for model training. Based on the AUC and multiple performance metrics in the training cohort, RF was identified as the optimal model (**Figure 4A**). Utilizing microbial biomarkers previously identified via LEfSe analysis, we constructed immunotherapy response prediction models at both the genus and species levels. The genus-level model, built on four key marker genera, achieved an AUC of 0.79 (**Figure 4B**). At the species level, the model incorporating seven differential species, including *B. caccae*, reached an AUC of 0.82 (**Figure 4C**). Given that all whole-genome shotgun (WGS) sequencing data were included in the training phase to maximize the sample size, we externally validated the genus-level model using an independent 16S rRNA sequencing dataset, resulting in an AUC of 0.61 (**Figure 4B**).

### 3.5 Uncovering key species affecting immunotherapy prognosis and stabilizing biomarkers

**Figure 5:**
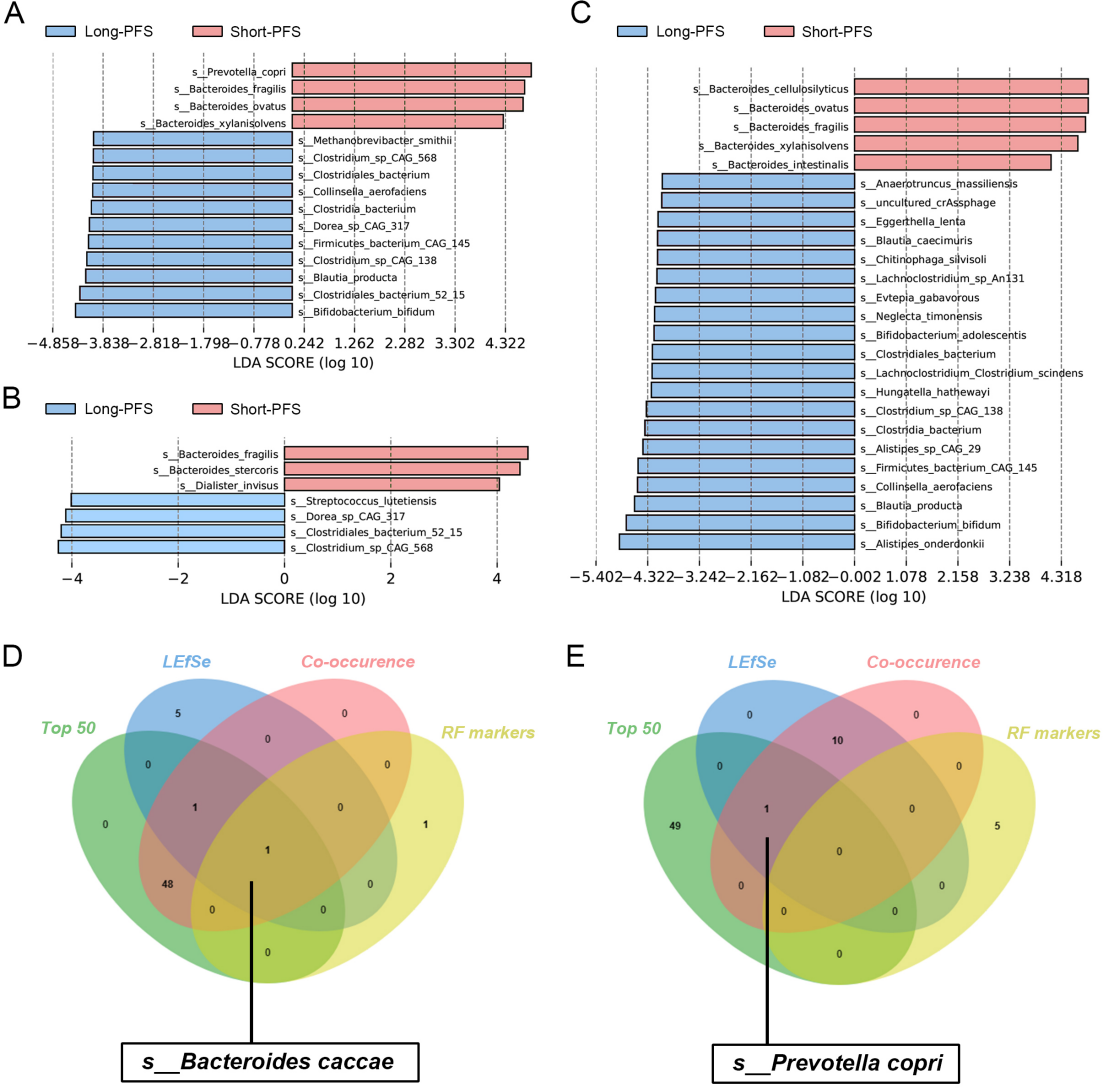
Key species affecting immunotherapy prognosis and stabilizing biomarkers. **(A)** To explore the difference of microbiota between the long PFS and short PFS groups based on the total population. **(B)** In the absence of antibiotic interference, the difference of gut microbiota between the long and short PFS groups. **(C)** Difference of microbiota between the two groups when they experienced antibiotic intervention. **(D)** Biomarkers that remained stable in the response group under the results of various bioinformatics analyses. **(E)** Biomarkers that remained stable in the non-responder group across various bioassays.

To investigate gut microbial species potentially associated with prognosis, we performed LEfSe analysis on two extreme patient cohorts: responders with PFS exceeding 6 months were classified as the long-PFS group, while non-responders with PFS under 3 months were classified as the short-PFS group. The analysis revealed that, without considering ATB exposure, *P. copri* was consistently identified as the top biomarker in the short-PFS group (**Figure 5A**). Conversely, *s_Bifidobacterium longum* was identified as a key biomarker for the long-PFS group across both the total cohort and the ATB-exposed subgroup (**Figures 5A, C**), corroborating previous findings of its significant enrichment in CAR-T cell therapy patients with prolonged PFS (Stein-Thoeringer et al., 2023). Notably, antibiotic exposure increased the number of differentially abundant species between groups (**Figure 5C**).

By integrating multi-dimensional analyses including abundance, differential significance, and intra-community interactions, we identified *B. caccae* as a robust biomarker consistently associated with the responder group, whereas *P. copri* was characteristic of the non-responder group (**Figures 5D, E**). Importantly, antibiotic exposure did not significantly affect these classifications (**Supplementary Figure S4**).

### 3.6 Dynamic changes of gut microbiota during immunotherapy

**Figure 6:**
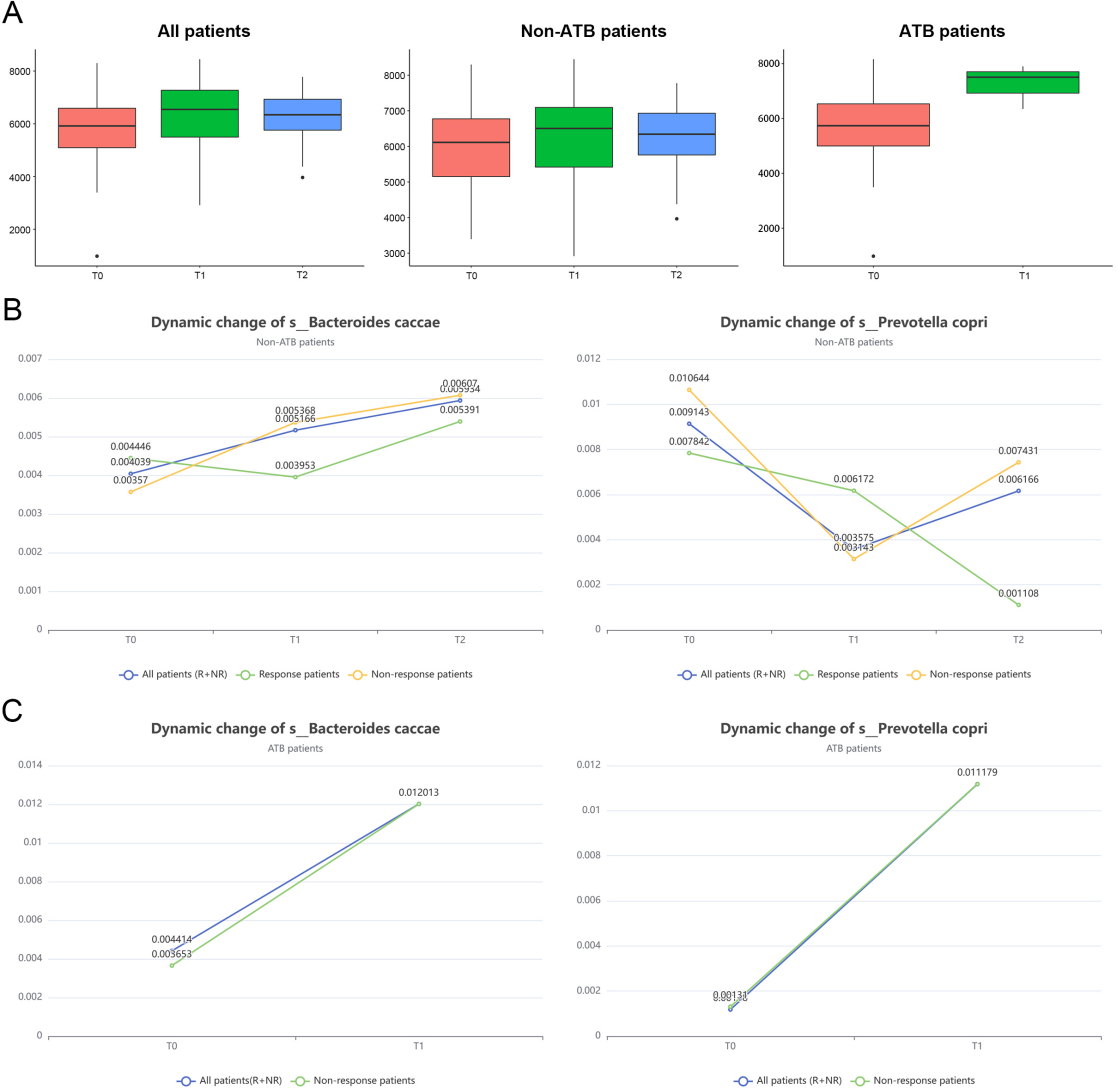
Changes in bacterial diversity and marker abundance during immunotherapy. **(A)** To investigate the changes in alpha diversity of gut microbiota at different time points after immunotherapy and the effect of antibiotics on this trend. **(B)** Changes of s_Bacteroides caccae in the response group at different time points after immunotherapy. **(C)** Changes in the marker s_Prevotella copri in the non-response group at different time points after immunotherapy.

As shown in **Figure 6A**, gut microbial α-diversity rebounded following the completion of the first stage of immunotherapy in all patients, with a more pronounced recovery observed in those who had received ATB. Interestingly, non-responders exhibited a greater increase in microbial diversity compared to responders (**Supplementary Figures S5A, B**), further supporting the notion that reductions in microbial diversity are closely associated with diminished immunotherapeutic efficacy. The subsequent decline in treatment response may also be linked to a secondary loss of microbial diversity.

Furthermore, we examined the dynamic changes in the abundance of two key microbial biomarkers across different phases of immunotherapy. In patients without prior ATB exposure, the abundance of the core responder species *B. caccae* increased significantly in both the R and NR groups following treatment, whereas the potentially detrimental taxon *P. copri* markedly decreased (**Figure 6B**). These findings suggest that restoration of beneficial microbes and suppression of harmful taxa may contribute to modulating host immune status and enhancing treatment responsiveness. However, due to the lack of dynamic data on host immune parameters and inflammatory markers in this study, it remains unclear whether these microbial shifts represent a causal factor for immunotherapy efficacy or a secondary outcome of treatment response.

Notably, in patients who had received prior ATB treatment, an initial broad depletion of GM was observed. During subsequent immunotherapy, a substantial rebound in the abundance of both biomarkers was detected (**Figure 6C**). This non-selective recovery may have led to the concurrent enrichment of both beneficial and harmful taxa, potentially with distinct kinetics. Such microbial imbalance could disrupt gut ecological homeostasis and impair the regulatory role of the microbiota in host immune modulation. This dysbiosis may partially explain the reduced efficacy of immunotherapy observed in the ATB-treated group.

### 3.7 Functional analysis of immune-related microbiota

**Figure 7:**
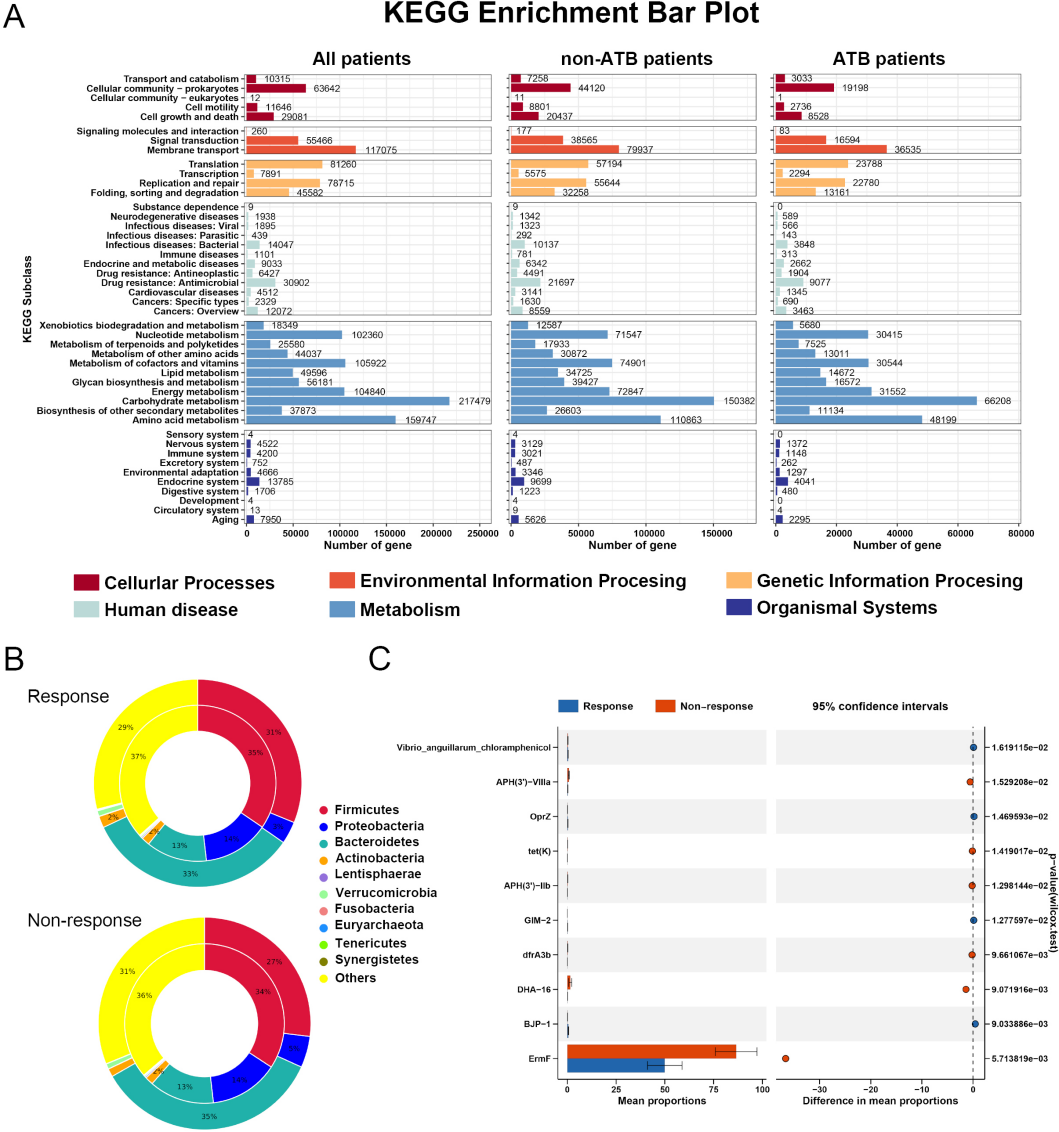
Functional analysis of gut microbiota in patients receiving immunotherapy. **(A)** A level 2 functional pathway statistical map was drawn based on KEGG annotation results. **(B)** Species attribution analysis of resistance genes between responders and non-responders when the effect of antibiotics was not considered **(C)** results of differential resistance genes between the two groups of patients.

The results of the KEGG functional enrichment analysis revealed that the GM were predominantly involved in metabolic pathways, with carbohydrate metabolism and amino acid metabolism being the most significantly enriched categories. Notably, this pattern remained consistent regardless of ATB exposure (**Figure 7A**). These findings further support the characterization of LC as a metabolism-associated disease. The results of subsequent differential analyses also indicated that the primary functional discrepancies between responders and non-responders were concentrated at the metabolic level (**Supplementary Figures S6**-**S8**), suggesting that modulating microbial metabolic functions may influence host metabolite profiles and ultimately affect immunotherapeutic outcomes.

In addition, we explored the association between the microbiome and antibiotic resistance genes using the CARD. Taxonomic assignment revealed that the majority of resistance genes in both responders and non-responders were affiliated with the phyla *Bacteroidetes* and *Proteobacteria*, and this distribution was not affected by ATB exposure (**Figure 7B**, **Supplementary Figures S7B**, **S8B**). Several resistance genes, including *ErmF* and the *rpoB* gene from *Bifidobacterium adolescentis*, exhibited significant intergroup differences. Notably, the administration of ATB further exacerbated the divergence in resistance gene profiles between patient groups (**Figure 7C**, **Supplementary Figures S7C**, **S8C**).

## 4 Discussion

In this meta-analysis, we conducted the largest metagenomic study to date focusing on the gut microbiome in the context of immunotherapy for LC. Participants were recruited from five independent cohorts across the globe, aiming to establish a broader and more reproducible microbiome signature associated with response to ICIs.

Overall, the responders exhibited higher species richness. Although this trend did not reach statistical significance—likely due to inter-cohort heterogeneity—it aligns with previously reported findings from both Chinese and US cohorts (Heshiki et al., 2020; Lee et al., 2021). The observed decrease in α-diversity among non-responders suggests a more profound dysbiosis in this group. Similar patterns of reduced α-diversity have been reported in various metabolic disorders and are closely associated with disease states (Dang and Marsland, 2019; Hakozaki et al., 2020). However, findings across studies remain inconsistent—for instance, in the study by Lee et al., non-responders demonstrated greater microbial diversity, whereas David et al. found no significant differences in α-diversity between responders and non-responders (Lee et al., 2021; Dora et al., 2023). Although reduced α-diversity has been proposed as a characteristic feature in melanoma immunotherapy, its relevance as a predictive biomarker for ICI efficacy in lung cancer remains uncertain and warrants further validation using real-world datasets.

At the taxonomic level, intergroup differences were primarily observed at the species level, and the use of ATBs appeared to influence the abundance of specific key taxa. Regarding the debated enrichment status of the “signature” bacterium *AKK*, our study demonstrated that *AKK* was relatively enriched in responders across both genus and species levels in the overall population and the ATB-exposed subgroup. In contrast, in participants who had not received ATBs, *AKK* was enriched in non-responders. These findings suggest that, in responders, ATBs may exert a stronger suppressive effect on non-*AKK* taxa—particularly those associated with non-response—thereby indirectly enhancing the relative abundance of AKK. Consequently, a key question for future clinical research is whether targeted antibiotic interventions can modulate the abundance balance between responder- and non-responder-associated microbiota to improve the efficacy of immunotherapy. This represents an important direction for mechanistic and translational investigation.

The R-specific strain *B. caccae* and the NR-specific strain *P. copri* identified in our study have been previously reported in the context of cancer immunotherapy. Both *B. caccae* and *AKK* exhibit ecological variability and have been associated with favorable outcomes in response to ICIs (Liu et al., 2023). Enrichment of *B. caccae* has also been observed in responders with melanoma (Frankel et al., 2017). A systematic review by Mariam et al., encompassing 18 studies across five types of solid tumors, reached a similar conclusion—*B. caccae* was enriched in patients with better prognoses. Whether *B. caccae* produces known immunomodulatory metabolites remains unknown. Future research should employ metabolomics, along with *in vitro* co-culture systems or organoid models, to uncover potential immune-regulatory mechanisms associated with this strain. In contrast, the pathogenic potential of *P. copri* has been more extensively studied. Xiang et al. demonstrated that GM transplantation from patients harboring *P. copri* induced severe inflammation and immune dysregulation in murine models (Qian et al., 2022). Furthermore, *P. copri* has been implicated in the pathogenesis of carboplatin-induced intestinal mucositis, and the abundance of the *Prevotella* genus has been linked to tumor invasiveness in melanoma (Yu et al., 2019; Vitali et al., 2022). Despite these pro-inflammatory and pro-tumorigenic associations, some studies have reported a higher abundance of *P. copri* in responders (Jin et al., 2019; Robinson et al., 2023). Whether this strain contributes to limiting the long-term benefits of ICI therapy remains an open question, requiring further investigation through mechanistic and *in vivo* studies.

In recent years, numerous high-impact studies have demonstrated the feasibility and clinical potential of microbiome-based predictive models for therapeutic response—for instance, Huang et al. developed a cross-cohort, pan-cancer immune response prediction model achieving an AUC of up to 0.89 (Huang et al., 2023). A more recent study published in *Nature Medicine* further confirmed that strain-level abundance differences derived from metagenomic approaches can be utilized to predict immunotherapy response across multiple cancer types (Gunjur et al., 2024). In the context of LC, existing models primarily focus on distinguishing patients with long versus short PFS. Notably, Liu et al. reported that models based on differential microbial taxa yielded suboptimal performance, whereas those leveraging microbial functional protein profiles achieved superior predictive accuracy, with an AUC of 0.95 (Liu et al., 2022). In addition, Lisa et al. introduced a threshold-based stratification using the abundance of *Akk*, defining patients as positive or negative subgroups, further highlighting the potential of this species in predicting immunotherapy response (Derosa et al., 2022).

Although models constructed using WGS sequencing data exhibit robust classification performance, our approach faced limitations during external validation. To maximize data utilization, all available WGS samples were included in the training set. Consequently, an independent validation cohort at the genus level was retrieved through systematic data mining, relying on 16S rRNA sequencing data. Due to inherent differences in taxonomic resolution and annotation depth between WGS and 16S platforms, the model’s AUC dropped to 0.61 in the 16S cohort, suggesting limited cross-platform generalizability. Nonetheless, this result remains informative, especially in the absence of large-scale independent WGS cohorts. Moving forward, we will continue monitoring the release of new WGS datasets and explore the integration of multi-omics data, including metagenomics, metabolomics, and transcriptomics, to enhance model robustness and cross-platform adaptability. As global, multi-center metagenomic datasets continue to grow, we anticipate further validation and refinement of our findings and models in broader population contexts.

KEGG-based functional profiling revealed a significant enrichment of metabolism-related pathways across all three groups, suggesting a common microbial contribution to metabolic reprogramming associated with tumor progression and survival (Li and Zhang, 2016). Notably, in immunotherapy responders, pathways involved in amino acid biosynthesis (e.g., tryptophan and branched-chain amino acids) and carbohydrate metabolism (e.g., glycolysis and the pentose phosphate pathway) were markedly upregulated (highlighted in orange bars), indicating a more active microbial role in metabolite production and energy provision (Tufail et al., 2024). Importantly, several microbial-derived metabolites—such as short-chain fatty acids and tryptophan derivatives—are known immunomodulatory agents that can enhance T cell activation or suppress pro-inflammatory signaling, potentially promoting improved therapeutic outcomes. In contrast, non-responders exhibited a significant upregulation of cancer-associated pathways, particularly those involved in PD-L1 expression and PD-1 checkpoint signaling, suggesting that certain microbial taxa may facilitate immune evasion mechanisms. These findings underscore the potential of leveraging microbial functional signatures to identify key taxa whose targeted modulation may reduce tumor immune escape and enhance immunotherapy responsiveness.

Our findings are highly consistent with a recent metatranscriptomic study conducted in a LC immunotherapy cohort, collectively highlighting the pivotal role of GM in modulating responses to immune checkpoint blockade (Dora et al., 2024). Both studies reported significantly higher gut microbial diversity in responders compared to non-responders, suggesting a potential link between microbial ecological complexity and enhanced therapeutic efficacy. Moreover, the consistent enrichment of *B. caccae* and *Akk* in responders across studies reinforces the stability and generalizability of *B. caccae* as a potential predictive biomarker. Future efforts should prioritize integrative multi-omics approaches—including metagenomics, metatranscriptomics, and metabolomics—to elucidate the mechanistic underpinnings of microbe-mediated immune modulation, ultimately advancing personalized prediction and clinical application of immunotherapy.

In addition, we constructed microbial co-occurrence networks to preliminarily investigate potential microbe–microbe interactions associated with immune response, aiming to identify key taxa that may exert antagonistic or synergistic effects in responders. Given the large number of annotated species in the dataset, our analysis was restricted to the top 50 most abundant taxa to enhance robustness. However, it should be noted that correlation-based co-occurrence network methods are inherently limited by the compositionality and sparsity of microbiome data, which may introduce biases in inferring microbial associations. Moreover, since the interactions identified through co-occurrence analysis have not yet been validated by independent analytical approaches, we did not incorporate these results into the final biomarker prediction model to maintain scientific rigor. Future studies may benefit from applying more advanced microbial network inference tools, such as SPIEC-EASI, CoNet, and FlashWeave, to improve the accuracy of interaction inference and enhance biological interpretability.

With the growing body of evidence linking the gut microbiome to cancer immunotherapy outcomes, microbiome profiling is emerging as a promising avenue to advance personalized immunotherapy in lung cancer, with considerable translational potential. First, specific microbial biomarkers—such as *B. caccae* and *Akk*—may be assessed through non-invasive fecal testing prior to ICI administration, enabling more accurate patient stratification, enhancing treatment response rates, and reducing unnecessary exposure for likely non-responders (Chen et al., 2019; Han et al., 2023). Second, GM data can inform more rational antibiotic stewardship, including optimization of timing, drug selection, and withdrawal windows, in order to preserve immunologically relevant commensal taxa. Finally, microbiome-informed personalized interventions—such as targeted probiotic supplementation, FMT, or microbial metabolite administration—offer a novel adjunctive strategy to improve ICI efficacy (Lu et al., 2022; Yadegar et al., 2024). However, these approaches must be pursued with rigorous safety evaluation and mechanistic validation. Collectively, these considerations establish a feasible framework for integrating microbiome analysis into the clinical management of lung cancer and advancing precision immuno-oncology.

In future studies, we will focus on the key gut microbial taxa identified in this work to investigate their roles in modulating antitumor immunity. Using *in vitro* co-culture systems, tumor organoid models, and humanized mouse models, we will systematically explore three mechanistic dimensions: microbial metabolite production, immunomodulatory capacity, and mucosal adhesion or translocation properties. To elucidate functional links between the microbiota and host immunity, we will integrate fecal metabolomic and serum immune transcriptomic data to reconstruct microbe–metabolite–immune signaling networks. This approach will help identify key metabolic mediators driving systemic immune responses and reveal potential causal axes underlying microbiota–host interactions. Additionally, we will expand real-world LC cohorts, particularly those with WGS data, to enable strain-level microbiome profiling and robust external validation across diverse populations stratified by ethnicity, age, and treatment background. Finally, we will integrate microbiome features with clinical parameters, immune phenotypes, and genomic alterations to build high-resolution, multi-dimensional models for predicting immunotherapy response.

## 5 Conclusion

We established the first global cross-cohort gut microbiota landscape associated with immunotherapy response and developed a machine learning-based predictive model with potential translational relevance. These findings offer new insights into the design of microbiome-informed immunotherapeutic strategies and the identification of novel adjuvants or therapeutic targets.

## Data Availability

The original contributions presented in the study are included in the article/**Supplementary Material**. Further inquiries can be directed to the corresponding author.
