# Supplementary material for: Exploring fecal microbiota signatures associated with immune response and antibiotic impact in NSCLC: insights from metagenomic and machine learning approaches
Source: Front Cell Infect Microbiol. 2025 Jul 28;15:1591076. doi: 10.3389/fcimb.2025.1591076 (PMC12336193; doi:10.3389/fcimb.2025.1591076)
Supplement: Supplementary file 1 [file Table1.docx]

**Exploring Fecal Microbiota Signatures Associated with Immune Response and Antibiotic Impact in NSCLC: Insights from Metagenomic and Machine Learning Approaches**

Wenjie Han^1,2^, Yuhang Zhou^1,2^, Yiwen Wang^1,2^, Xiaolin Liu^3^, Tao Sun^1,4^ and Junnan Xu^1,2,4*^

**Content**

[1 Strategy for Data Mining and Cohort Enrollment Criteria 2](#_Toc21471)

[1.1 Electronic search strategy in PubMed and Web of Science databases 2](#_Toc25027)

[1.2 PRISMA Flowchart of the Data Retrieval and Screening Process 2](#_Toc3474)

[1.3 Cohort Enrollment Criteria 3](#_Toc1056)

[2 Quality Assessment and Risk of Bias Evaluation 4](#_Toc818)

[2.1 Quality assessment results of the included studies with the ROBIS tool. 4](#_Toc11196)

[2.2 Quality assessment results of the included studies using the Joanna Briggs Institute Critical Appraisal Checklist for Case-Control Studies. 5](#_Toc5903)

[3 Supplementary Figures 7](#_Toc25524)

[4 Supplementary Tables 17](#_Toc22937)

# Strategy for Data Mining and Cohort Enrollment Criteria

## Electronic search strategy in PubMed and Web of Science databases

**MEDLINE (PUBMED)**

1. (gastrointestinal OR intestinal OR fecal OR fecal OR stool OR respiratory ) [tiab]
2. (microbiome OR microbiota OR ecosystem OR bacteria OR flora OR microflora OR dysbiosis) [tiab]
3. ("lung cancer" OR 'lung neoplasm" OR "pulmonary neoplasm" OR "pulmonary cancer' OR "lung adenocarcinoma" OR "Squamous cell lung carcinoma") AND（cancer or tumor or carcinoma）[tiab]
4. (Immunity OR Immunotherapy OR Programmed Death-1 OR Programmed Death-L1 )[tiab]
5. (Antibiotics OR Antimicrobial therapy OR Antibiotic therapy)[tiab]
6. (Metagenomic OR 16s rRNA OR transcriptome OR meta-analysis) [tiab]
7. 1 AND 2 AND 3 AND 4 AND 5 AND 6

**Web of Science**

1. (gastrointestinal OR intestinal OR fecal OR fecal OR stool OR respiratory ) [tiab]
2. (microbiome OR microbiota OR ecosystem OR bacteria OR flora OR microflora OR dysbiosis) [tiab]
3. ("lung cancer" OR 'lung neoplasm" OR "pulmonary neoplasm" OR "pulmonary cancer' OR "lung adenocarcinoma" OR "Squamous cell lung carcinoma") AND（cancer or tumor or carcinoma）[tiab]
4. (Immunity OR Immunotherapy OR Programmed Death-1 OR Programmed Death-L1 )[tiab]
5. (Antibiotics OR Antimicrobial therapy OR Antibiotic therapy)[tiab]
6. (Metagenomic OR 16s rRNA OR transcriptome OR meta-analysis) [tiab]
7. 1 AND 2 AND 3 AND 4 AND 5 AND 6

## PRISMA Flowchart of the Data Retrieval and Screening Process


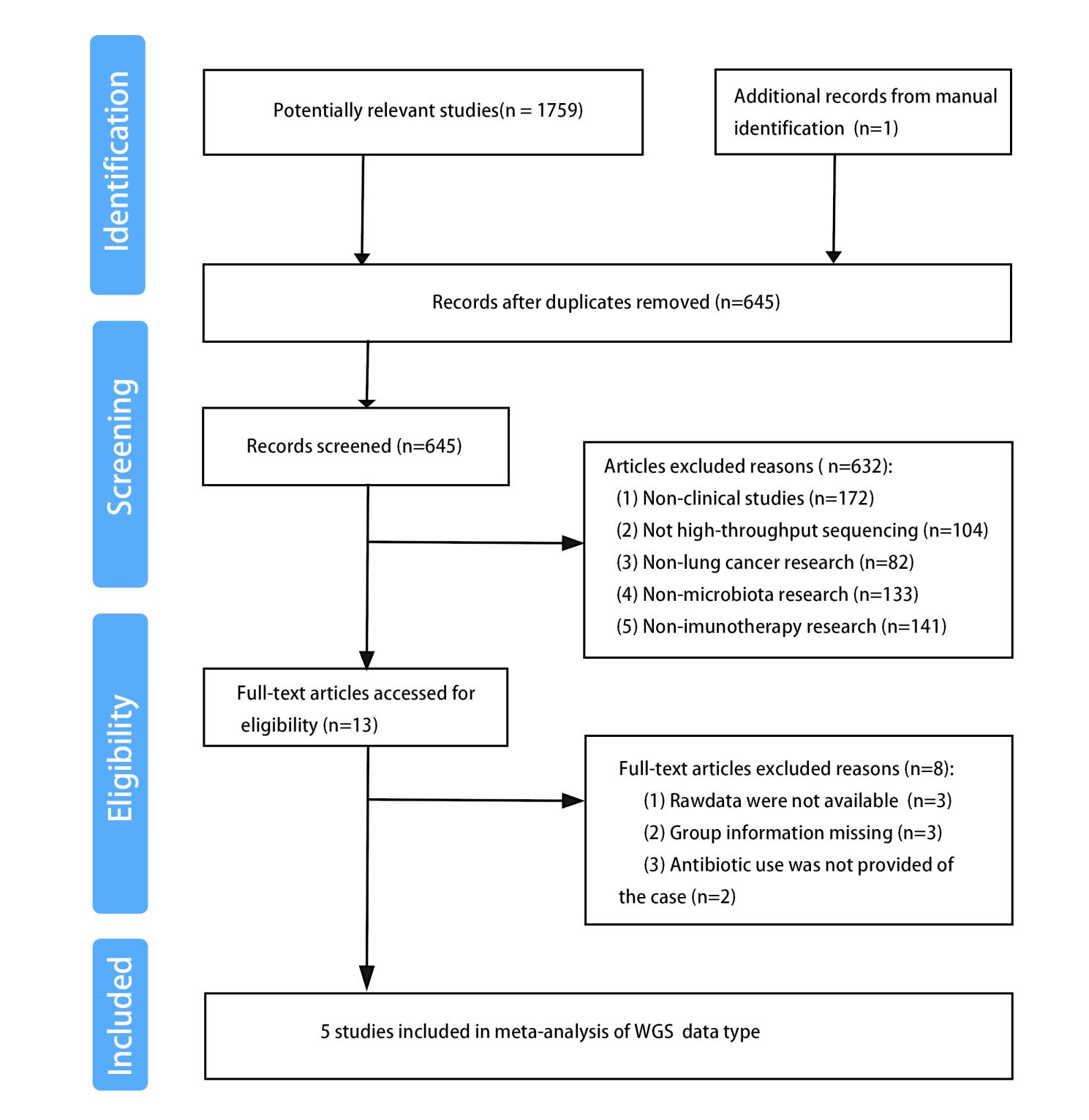


**Figure 1. Meta-analysis data mining PLASM flow chart.**

## Cohort Enrollment Criteria

Inclusion criteria were as follows: (1) histopathologically confirmed lung cancer (LC); (2) prior receipt of one or more systemic therapies before initiation of immunotherapy; (3) treatment with immune checkpoint inhibitor (ICI) monotherapy, including but not limited to pembrolizumab, nivolumab, or atezolizumab; (4) an expected survival of at least three months; (5) enrollment regardless of antibiotic (ATB) or probiotic administration within the three months preceding fecal sample collection. Subsequent analyses will stratify patients based on ATB exposure to evaluate its potential impact on immunotherapy outcomes; (6) fecal samples were longitudinally collected at baseline prior to immunotherapy initiation (T0), at one month (T1), and at two months (T2) post-treatment initiation.

All participants provided written informed consent prior to study inclusion. The study protocol was approved by the institutional ethics committee. Patients with inflammatory bowel disease or other acute or chronic gastrointestinal conditions were excluded.

To minimize bias resulting from heterogeneous definitions of treatment response across studies, individual patient clinical data were extracted from the original publications. Based on the REXIST1.1 criteria, patients were uniformly classified into responder groups (including stable disease, complete response, or partial response) and non-responder groups (progressive disease or death). Microbial abundance and functional analyses were performed using log-normalized unique gene (UniqGenes) datasets.

# Quality Assessment and Risk of Bias Evaluation

## Quality assessment results of the included studies with the ROBIS tool.

The summary of risk of bias assessment using the ROBIS tool is presented in Table 1, and the corresponding visualization is shown in Figure 1.

**Table 1. Quality assessment results of the included studies with the ROBIS tool.**

| **Review** | | | **Phase 1** | | | | **Phase 2** |
| --- | --- | --- | --- | --- | --- | --- | --- |
| **Disorder** | **Year** | **First Author** | **Study eligibility criteria** | **Identification and selection of studies** | **Data collection and study appraisal** | **Synthesis and findings** | **RISK OF BIAS IN THE REVIEW*** |
|  |  |  |  |  |  |  |  |
|  |  |  |  |  |  |  |  |
| NSCLC | 2022 | Ben Liu | low | low | low | low | low |
| NSCLC | 2021 | Se-Hoon Lee | low | low | unclear | high | high |
| LC, breast cancer, colon cancer, rectal cancer, pancreatic cancer, ovarian cancer, prostate cancer, and blood cancer | 2020 | Yoshitaro Heshiki | low | unclear | high | low | high |
|  |  |  |  |  |  |  |  |
| NSCLC and RCC | 2018 | Bertrand Routy | low | low | low | low | low |
| Healthy human | 2015 | HMP |  |  |  |  |  |
| NSCLC | 2021 | Se-Hoon Lee | unclear | unclear | low | low | unclear |
| NSCLC | 2022 | Rachel C. Newsome | low | low | low | low | low |

**Low** indicates a low risk of bias, **High** indicates a high risk of bias, and **Unclear** denotes insufficient information to determine the risk of bias.
*This domain is evaluated based on the following criteria:
A. Whether the interpretation of findings adequately addressed all concerns raised during the Phase 2 assessment;
B. Whether the relevance of the included studies to the review question was appropriately considered; and
C. Whether the reviewers avoided overemphasizing results based solely on statistical significance.

NSCLC, non-small cell lung cancer; LC，lung cancer；RCC，renal cell carcinoma; HMP, The Human Microbiome Project (HMP) is a large-scale DNA sequencing program led by the National Institutes of Health. From it, we extracted metagenomic shotgun sequencing data from healthy subjects for downstream analysis.(http://segatalab.cibio.unitn.it/tools/metaphlan2/)


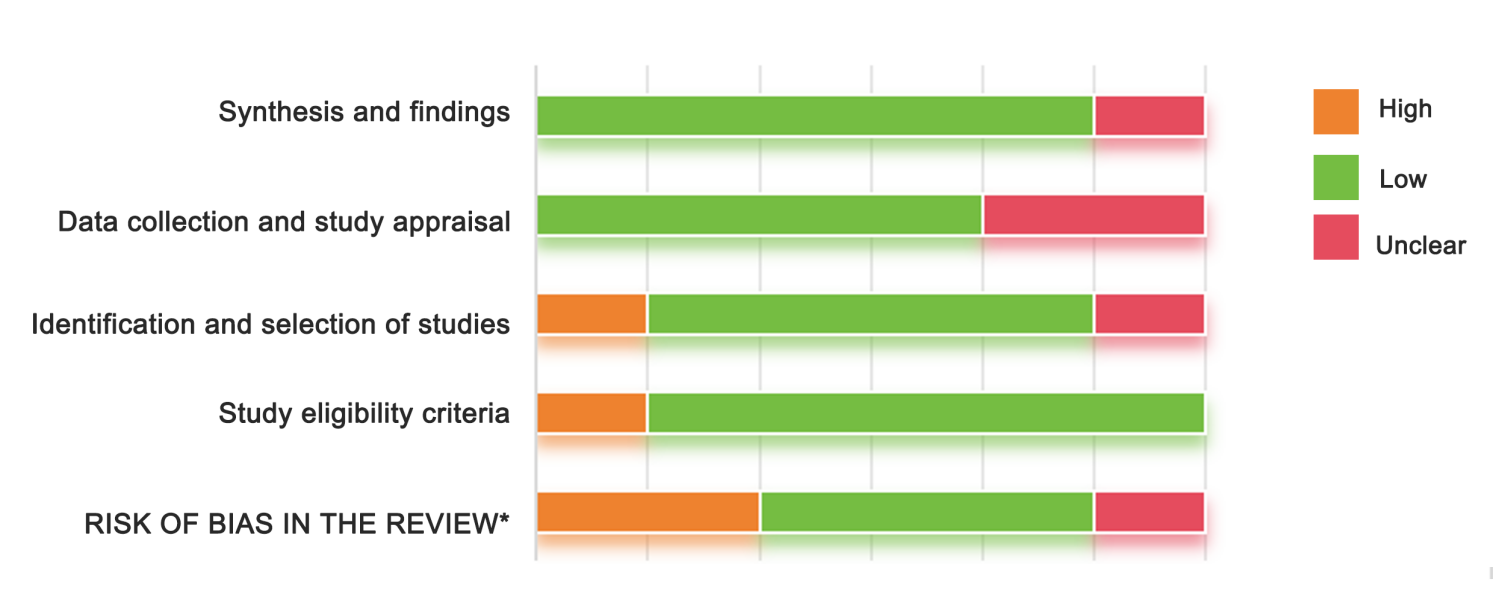


**Figure 1. Visualization of Risk of Bias Assessment Using the ROBIS Tool**

**Summary:** The overall risk of bias in the included systematic reviews was low (Table 1). The main limitation was that some studies did not adequately account for potential confounding factors. To address this issue, we conducted a heterogeneity assessment prior to data analysis, using the confounder analysis approach proposed by Jakob Wirbel et al. as a reference^[1]^.

1. Wirbel J, Pyl PT, Kartal E, Zych K, Kashani A, Milanese A, Fleck JS, Voigt AY, Palleja A, Ponnudurai R, Sunagawa S, Coelho LP, Schrotz-King P, Vogtmann E, Habermann N, Niméus E, Thomas AM, Manghi P, Gandini S, Serrano D, Mizutani S, Shiroma H, Shiba S, Shibata T, Yachida S, Yamada T, Waldron L, Naccarati A, Segata N, Sinha R, Ulrich CM, Brenner H, Arumugam M, Bork P, Zeller G. Meta-analysis of fecal metagenomes reveals global microbial signatures that are specific for colorectal cancer. Nat Med. 2019 Apr;25(4):679-689. doi: 10.1038/s41591-019-0406-6. Epub 2019 Apr 1. PMID: 30936547; PMCID: PMC7984229.

## Quality assessment results of the included studies using the Joanna Briggs Institute Critical Appraisal Checklist for Case-Control Studies.

**Table 2. Quality assessment results of the included studies using the Joanna Briggs Institute Critical Appraisal Checklist for Case-Control Studies.**

| **Study(First Author & Year)** | **1. Were the criteria for inclusion in the sample clearly defined?** | **2. Were the study subjects and the setting described in detail？** | **3. Was the exposure measured in a valid and reliable way？** | **4. Were objective, standard criteria used for measurement of the condition?** | **5. Were confounding factors identified?** | **6. Were the outcomes measured in a valid and reliable way?** | **7. Were cases and controls matched** | **8. Was appropriate statistical analysis used?** |
| --- | --- | --- | --- | --- | --- | --- | --- | --- |
|  |  |  |  |  |  |  | **appropriately?** |  |
| Ben Liu,2022 | Yes | Yes | Yes | Yes | Yes | Yes | Yes | Yes |
| Se-Hoon Lee,2021 | Yes | Yes | Yes | Yes | NO | Yes | Yes | Yes |
| Yoshitaro Heshiki,2020 | Yes | Yes | Yes | Yes | NO | Yes | Yes | Yes |
| Bertrand Routy,2018 | Yes | Yes | Yes | Yes | Yes | Yes | Yes | Yes |
| HMPb,2015 | Yes | Yes | Yes | Yes | NO | Yes | NO | Yes |
| Se-Hoon Lee,2021 | Yes | Yes | Yes | Yes | NO | Yes | Yes | Yes |
| Rachel C. Newsome,2022 | Yes | Yes | Yes | Yes | Yes | Yes | Yes | Yes |

# Supplementary Figures


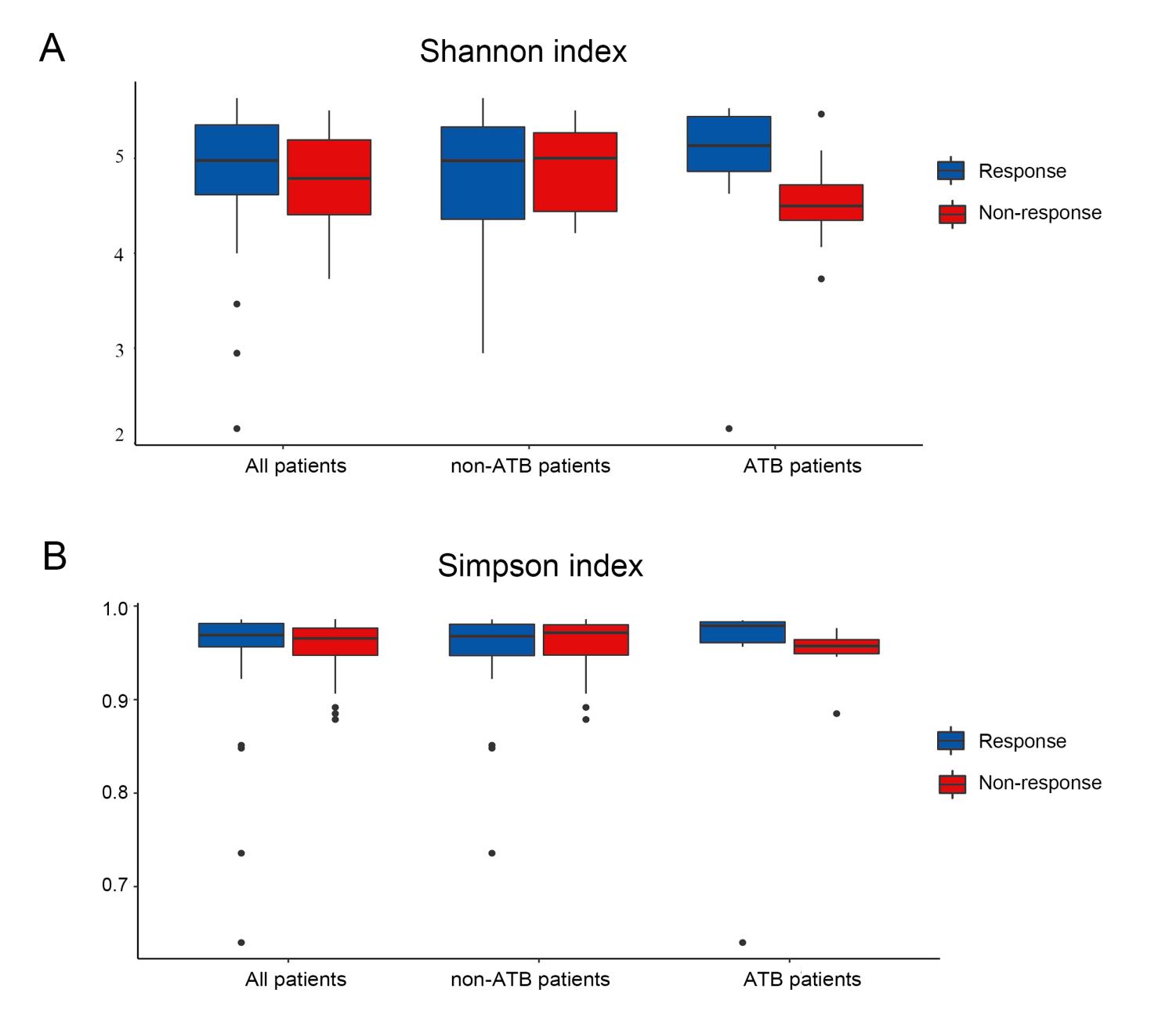


**Supplementary Figure 1**. Differences in alpha diversity between responders and non-responders (A: Shannon index; B: Simpson index) and the effect of antibiotics on this trend.


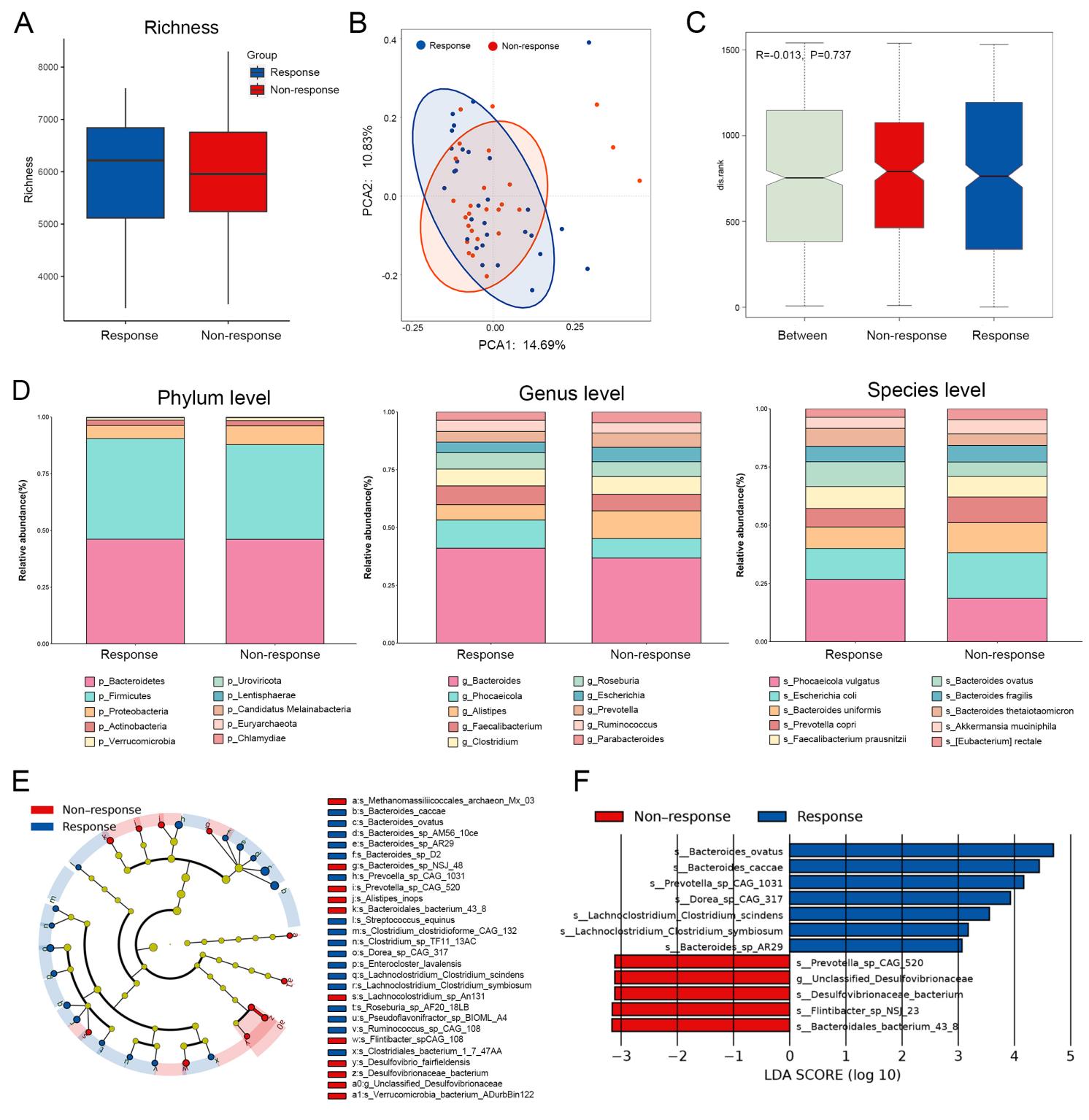


**Supplementary Figure 2**. When the interference of antibiotics was excluded, the composition of gut microbiota was different between responders and non-responders. (A) Richness was used to investigate the difference of α-diversity between the two groups; (B) PCOA analysis of gut microbiota in responders versus non-responders; (C) similarity analysis between the two groups; (D) Species composition differences at phylum, genus and species levels between the two groups; (E) Cladogram of bacterium with significant differences between the two groups; (F) Results of lefse analysis showed differences between the two groups.


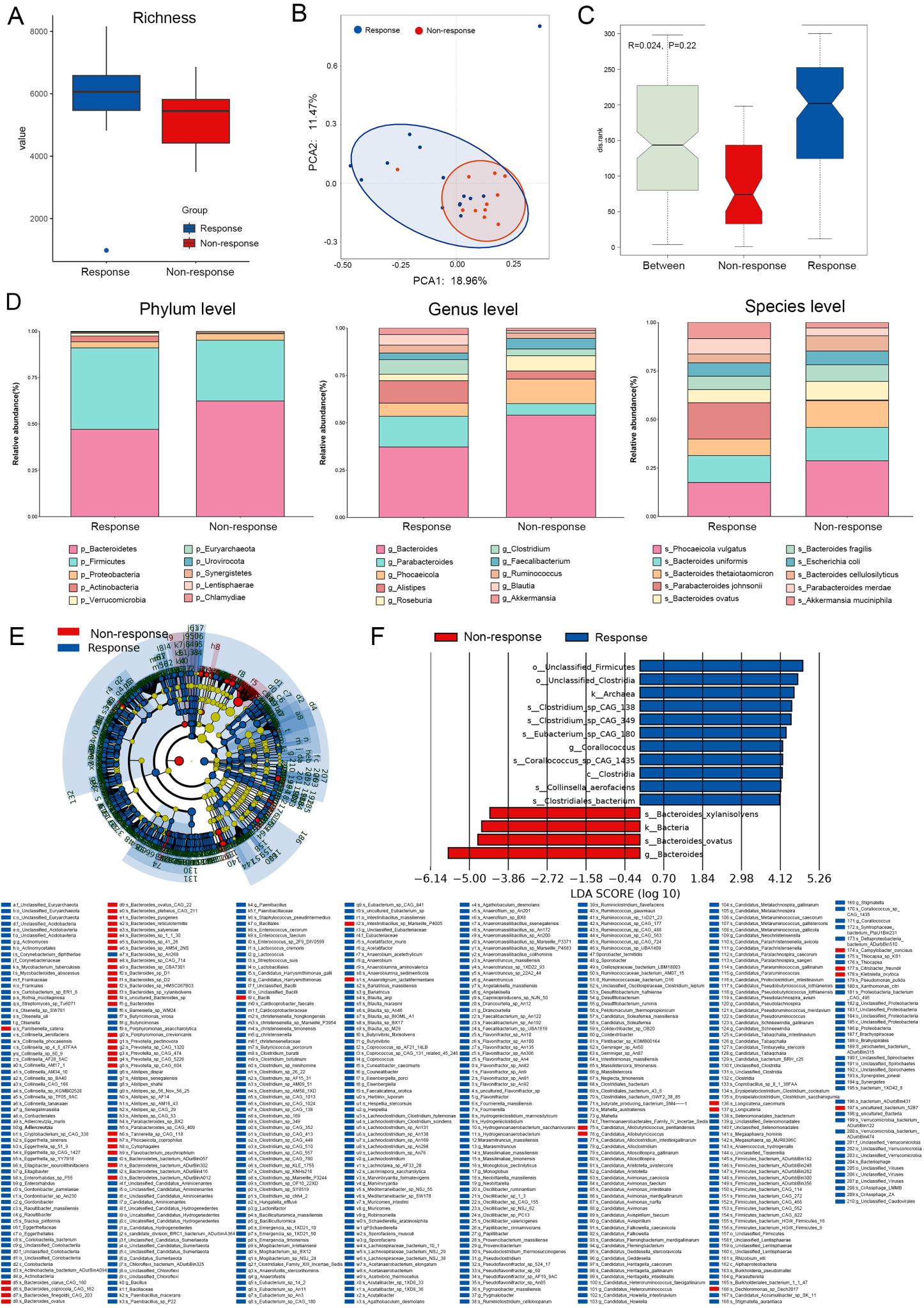


**Supplementary Figure 3**. After antibiotic intervention, the composition of gut microbiota is different between responders and non-responders. (A) Richness was used to investigate the difference of α-diversity between the two groups; (B) PCOA analysis of gut microbiota in responders versus non-responders; (C) similarity analysis between the two groups; (D) Species composition differences at phylum, genus and species levels between the two groups; (E) Cladogram of bacterium with significant differences between the two groups; (F) Results of lefse analysis showed differences between the two groups.


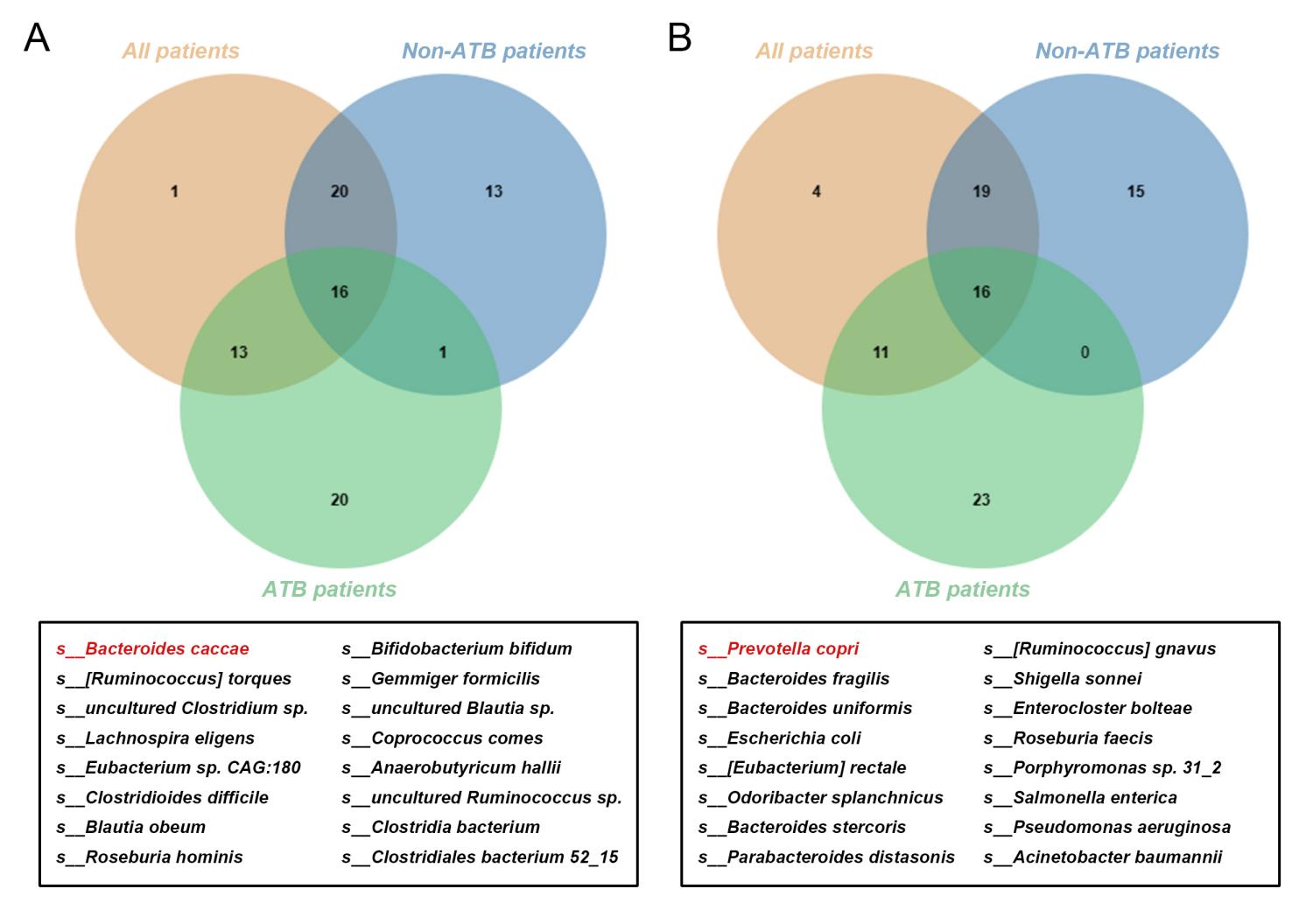


**Supplementary Figure 4**. Within top50, stably enriched bacteria without antibiotic interference. (A) Response group; (B) non-responder group.


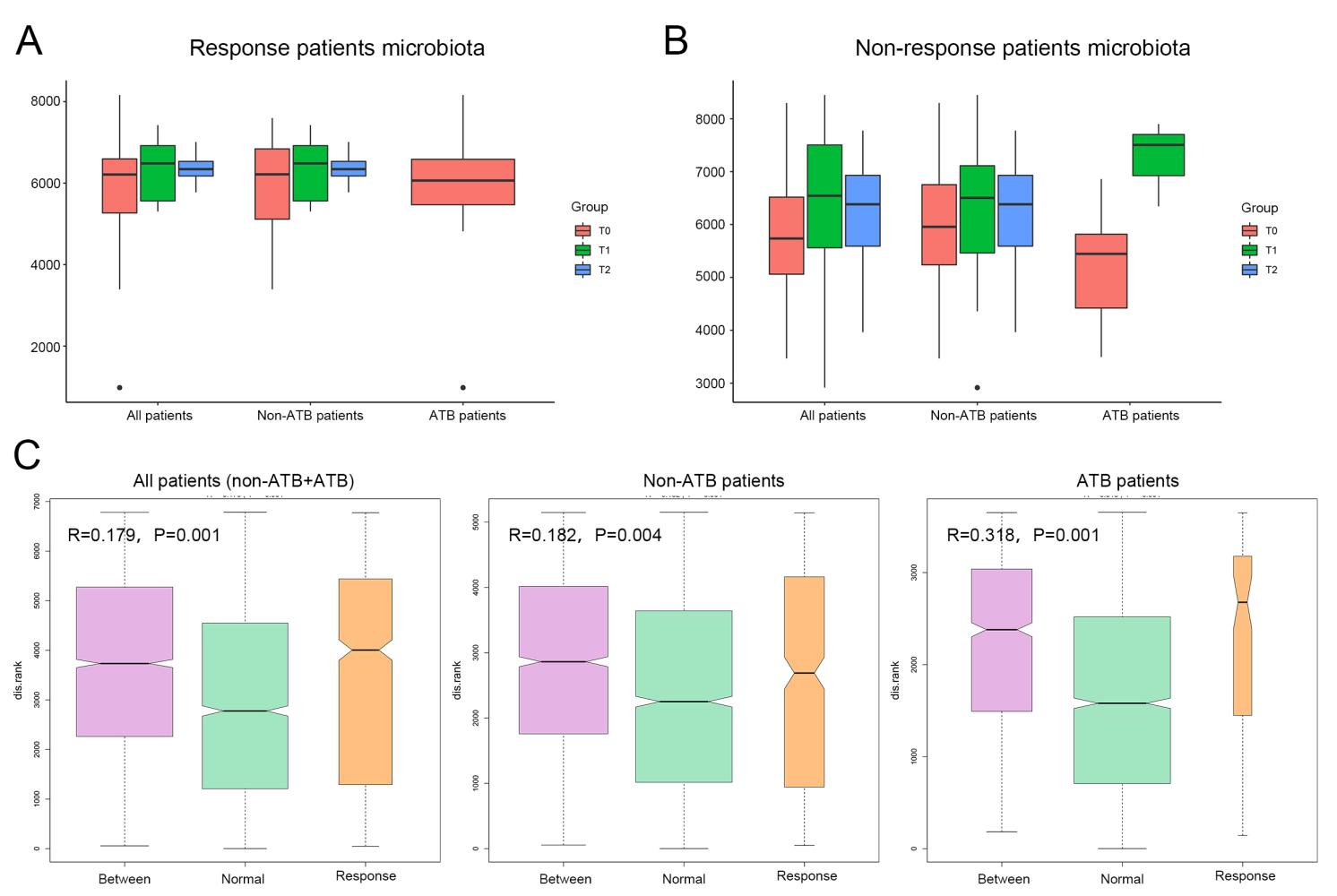


**Supplementary Figure 5**. (A) Changes in gut microbiota diversity at different time points after immunotherapy in responders; (B) Changes in gut microbiota diversity at different time points after immunotherapy in non-responsive patients; (C) To investigate the similarity of microbiota between responders and healthy subjects under different conditions.


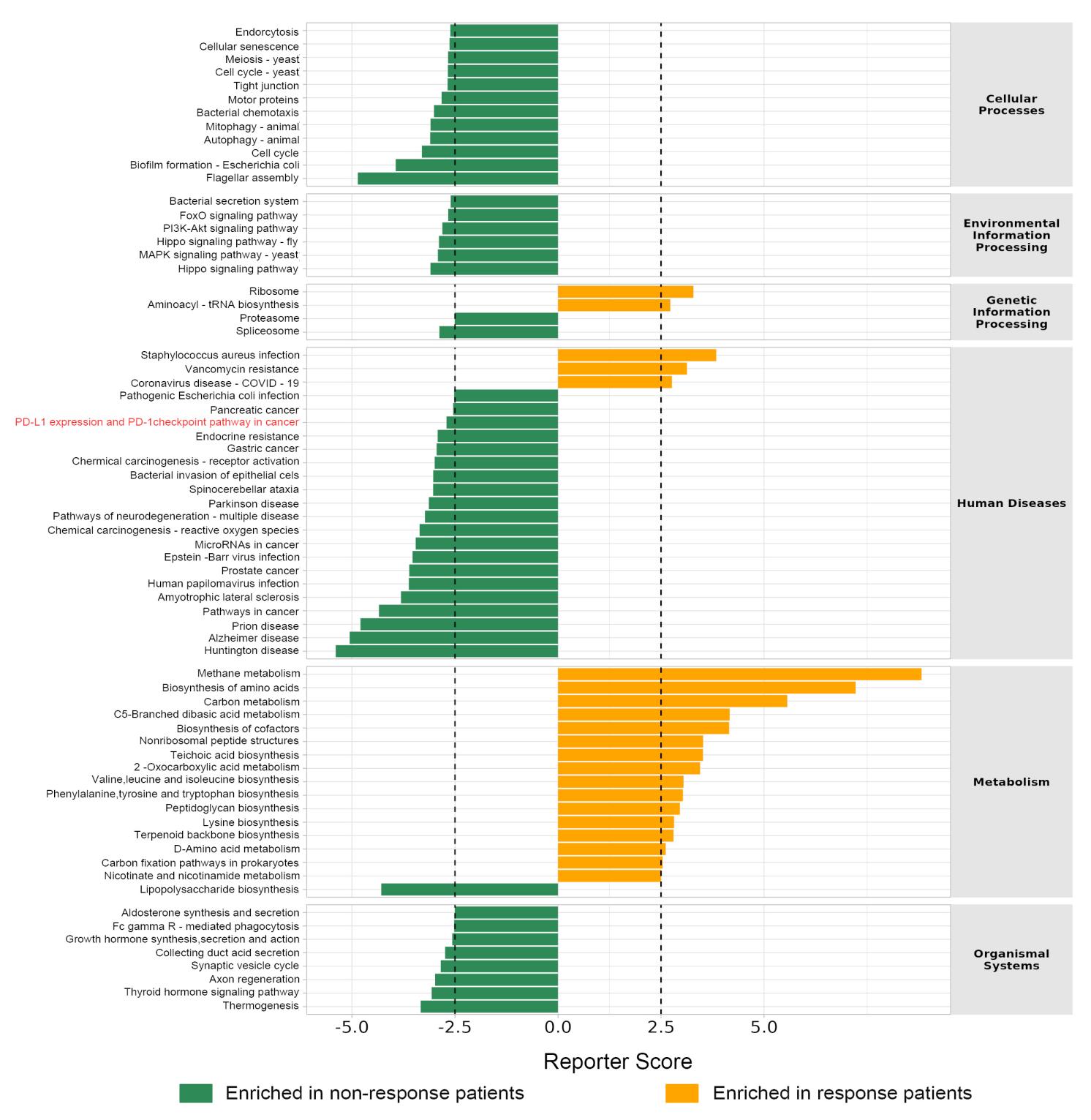


**Supplementary Figure 6**. Based on the total population, the functional differences of the microbiota between the response group and the non-response group were analyzed.


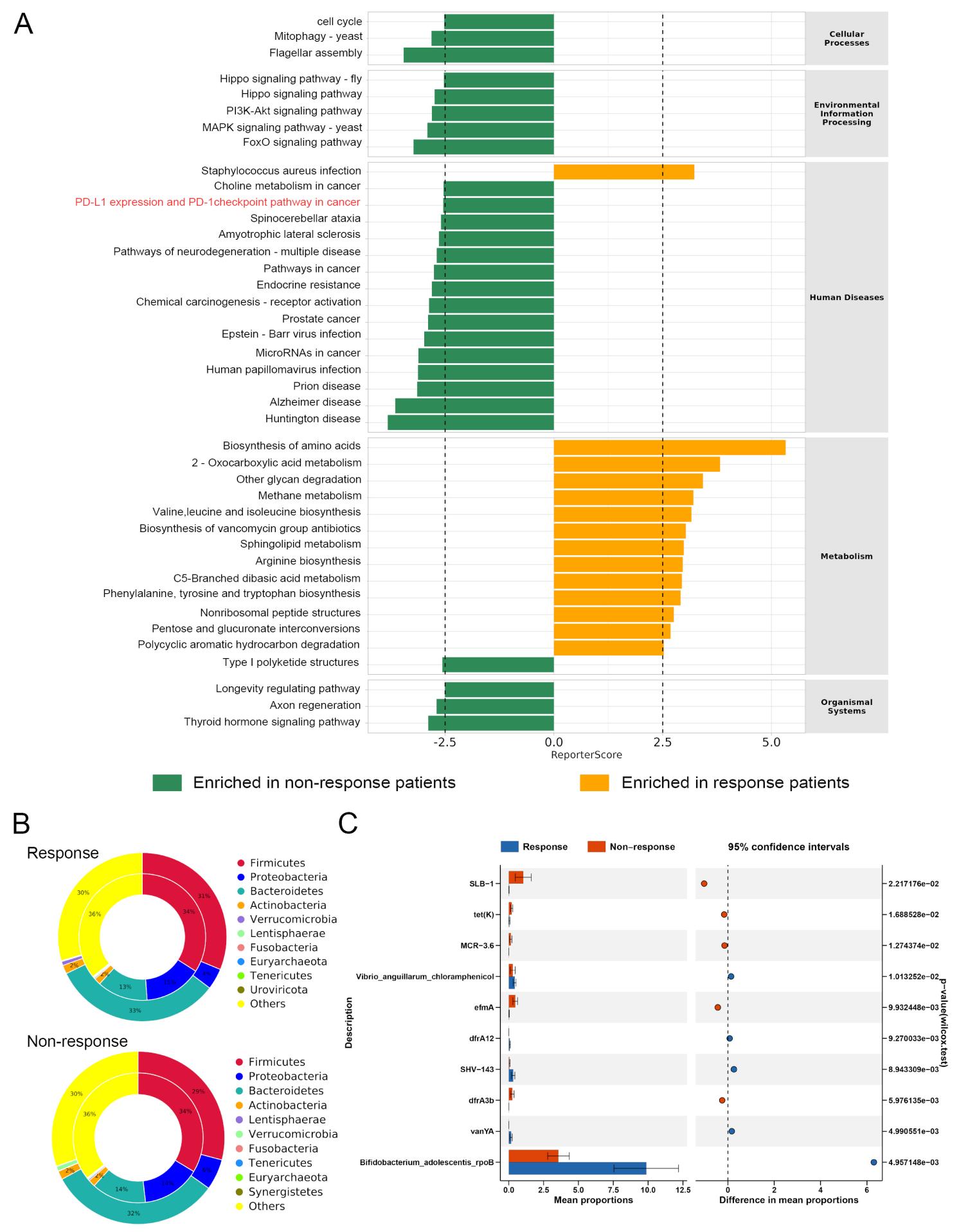


**Supplementary Figure 7**. Analysis of gut microbiota function in the non-ATB population. (A) Functional differences of gut microbiota between responders and non-responders; (B) Attribution analysis of resistance genes in the two groups of patients. (C) analysis of resistance gene differences between the two groups.


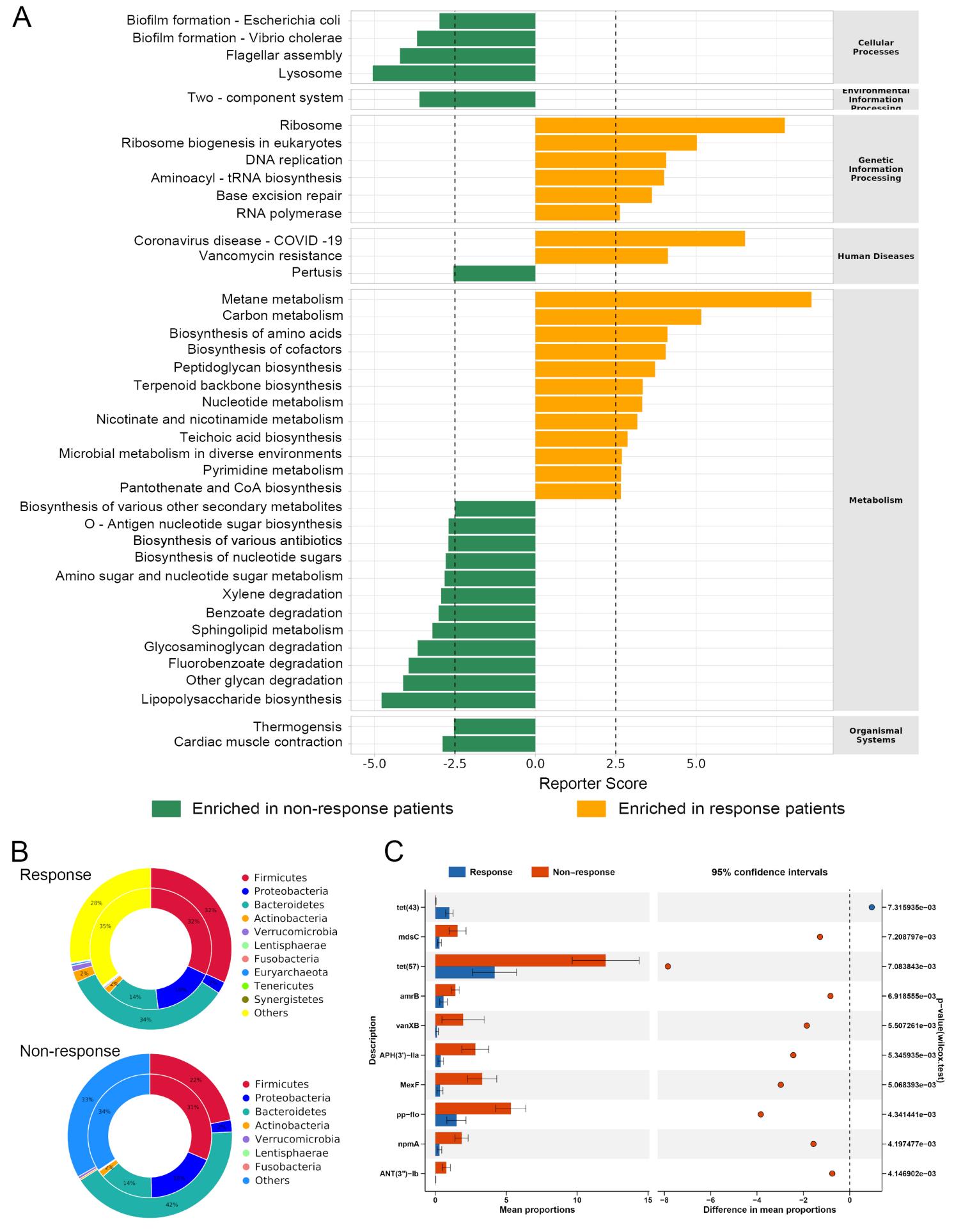


**Supplementary Figure 8**. Analysis of gut microbiota function in the ATB population. (A) Functional differences of gut microbiota between responders and non-responders; (B) Attribution analysis of resistance genes in the two groups of patients. (C) analysis of resistance gene differences between the two groups.

# Supplementary Tables

**Supplementary Table 1. Methodological summary of sample processing for each study**

| **Study** | **Sample type** | **Collection method** | **Long-term storage condition** | **DNA extraction method** | **Quantification of DNA concentration** | **amplicon lengths** | **platform** |
| --- | --- | --- | --- | --- | --- | --- | --- |
| Liu et al. | Fecal | using a Commode Specimen Collection System | stored at − 80 °C prior to processing | using DNeasy PowerLyzer PowerSoil Kit (Qiagen, Germantown, MD) | Qubit (Thermo Fisher Scientific, USA) | 240 and 260 bp | Illumina MiSeq |
| Lee et al. | Fecal | following detailed printed instructions | stored at −80 °C. | using a FastDNA SPIN kit for soil (MP Biomedicals) | using a NanoDrop One Spectrophotometer (Thermo Fisher Scientific). | 151-bp | Illumina HiSeq 4000 |
| Yoshitaro et al. | Fecal | _ | _ | _ | Library preparation (using KAPA Hyper Prep Kit KR0961-V1.14) and Illumina sequencing were done at the University of Hong Kong | 20 to 100 bp | Illumina HiSeq 1500 with PE100 |
|  |  |  |  |  |  |  |  |
|  |  |  |  |  |  |  |  |
| Bertrand et al. | Fecal | _ | _ | _ | _ | 150bp | _ |
| HMP* | Fecal | _ | _ | _ | _ | 101bp paired-end reads | Illumina GAIIx platform |
| Lee et al. | Fecal | following detailed printed instructions | stored at −80 °C. | using a FastDNA SPIN kit for soil (MP Biomedicals) | using a NanoDrop One Spectrophotometer (Thermo Fisher Scientific). | _ | Illumina |
| Rachel C.et al. | Fecal | Liquid Dental Transport  Medium (LDTM; Anaerobe Systems, Morgan Hill, CA)  stool collection kit | stored at −80 °C. | using the DNeasy 96 PowerSoil Pro  QIAcube HT (QIAGEN) | _ | _ | Illumina MiSeq |
|  |  |  |  |  |  |  |  |
|  |  |  |  |  |  |  |  |

*，HMP, human microbiome project, as provided by MetaPhlAn2 (http://segatalab.cibio.unitn.it/tools/metaphlan2/). This study characterized the microbiome composition of different body sites by 16s rRNA or whole metagenome shotgun (mWGS) sequencing.

**Supplement table 2. Summary of beta diversity and research methods across studies.**

| **Study** | **Year** | **Metric** | **Analysis** | **Test** | **Finding** |
| --- | --- | --- | --- | --- | --- |
| Liu et al. | 2022 | Bray-Curtis | PCoA | Kruskal–Wallis test | No sig. different |
| Lee et al. | 2021 | Unweighted UniFrac | PCA | Two-way analysis of variance | Sig. different |
| Yoshitaro et al. | 2020 | Bray-Curtis，, ANOSIM | PCoA | Wilcoxon rank-sum test | Sig. different |
| Bertrand et al. | 2018 | - | - | - | - |
| Rachel C.et al. | 2022 | unweighted and weighted UniFrac | PCA | a two-tailed Mann-Whitney U-test | Sig. different |

PCoA, principal coordinate analysis; ANOSIM, analysis of similarities; PCA, principal component analysis. The study grouping of Bertrand et al. is different from ours in that it does not perform a comparison of β-diversity
